# Bibliometric and network analysis of acute conus medullaris syndrome and conus-related complications after lumbar spine procedures

**DOI:** 10.3389/fsurg.2026.1806486

**Published:** 2026-05-20

**Authors:** Bilal Ibrahim, Abdallah Arabyat, Waleed F. Dabbas, Mohammad Hiasat, Qais Samara, Mustafa Nadi, Zohair A. Ibrahim, Abed Al Haleem Bqour

**Affiliations:** 1 Division of Neurosurgery, Department of Special Surgery, Al-Balqa Applied University, Al-Salt, Jordan; 2 Department of Neurosurgery, Neuron Clinics, Amman, Jordan; 3 College of Medicine, Jordan University of Science and Technology, Ar-Ramtha, Jordan; 4 Division of Neurosurgery, Al-Hussein Al-Salt New Hospital, Al-Salt, Jordan

**Keywords:** acute conus medullaris syndrome, bibliometric analysis, conus medullaris, lumbar discectomy, minimally invasive spine surgery, postoperative complications

## Abstract

**Background:**

Acute conus medullaris syndrome (ACMS) is an uncommon but potentially devastating neurological complication that may occur after lumbar spine procedures, including minimally invasive lumbar discectomy. Despite its very low incidence, ACMS can result in major functional impairment and significant medicolegal consequences. The available evidence is largely case-based and widely dispersed, and no bibliometric synthesis has comprehensively mapped the global research landscape on ACMS and related conus-level postoperative complications.

**Methods:**

We searched PubMed (MEDLINE), Scopus, the Cochrane Library, and JSTAGE (January 1977–February 2025). After de-duplication and screening, no additional unique eligible publications were identified beyond PubMed; the final corpus therefore comprised PubMed-indexed records retrieved using MeSH terms and free-text keywords. Study identification and selection were reported in accordance with PRISMA 2020, adapted for bibliometric/mapping review methodology. Records were analyzed using Bibliometrix (R/Biblioshiny) and VOSviewer to assess productivity, collaboration, source concentration, and thematic structures.

**Results:**

The final dataset included 46 publications (1977–2025) from 34 sources, authored by 185 authors, with two single-authored documents and a mean of 4.15 co-authors per document. International co-authorship was limited (4.35%). PubMed publication-type tagging indicated that 38 records (82.6%) carried a “case reports” tag and 11 records (23.9%) carried a “review” tag (tags may overlap within a single record). A small core of specialist spine and neurosurgical journals accounted for a disproportionate share of publications. Thematic mapping showed dominant technique- and outcome-focused themes, whereas ACMS did not consistently emerge as a mature standalone thematic cluster.

**Conclusions:**

The global literature on ACMS and conus-related postoperative complications after lumbar spine procedures is extremely sparse, fragmented, and predominantly case-based, with limited international collaboration. Given the small sample size (46 publications), our findings should be interpreted as exploratory rather than definitive. Advancing knowledge in this area will likely require multicentre registries, standardized reporting frameworks, and coordinated data-sharing to enable cumulative evidence generation and improved patient-safety learning.

## 1 Introduction

Acute conus medullaris syndrome (ACMS) is an uncommon but severe neurological complication that can occur after lumbar spine procedures, including minimally invasive techniques such as microdiscectomy and endoscopic discectomy. Clinically, ACMS is characterized by rapid-onset neurological deficits—including lower-limb motor weakness, sphincter dysfunction, and perineal sensory disturbance—and may result in permanent disability if not promptly recognized and managed (1, 2). Although the incidence is low relative to the global volume of lumbar discectomy procedures, the magnitude of functional impairment and the medicolegal consequences associated with missed or delayed diagnosis make ACMS an important patient-safety concern in spine surgery (3, 4).

Minimally invasive lumbar discectomy has been increasingly adopted over recent decades, largely because it is associated with reduced soft-tissue disruption, shorter hospitalization, and faster recovery compared with open approaches (5, 6). However, shifts in technique and instrumentation have been accompanied by the emergence of less common—and sometimes novel—complication patterns. Spinal dural arteriovenous fistulas (SDAVFs), epidural arteriovenous fistulas (EDAVFs), and other iatrogenic vascular injuries have been reported as rare but devastating causes of postoperative conus-level neurological deterioration, including ACMS (7–9). Contemporary reports emphasize that delayed recognition of these vascular lesions is associated with poorer neurological outcomes, reinforcing the need for heightened clinical awareness and timely postoperative imaging when unexplained conus-level deficits develop (10, 11).

Bibliometric approaches provide a useful framework for analyzing scattered, low-incidence literatures that are dominated by case reports and small series. Unlike systematic reviews or meta-analyses, which rely primarily on pooled clinical outcomes, bibliometric studies map scientific production, collaboration patterns, and knowledge structures using publication metadata and network analytics (12, 13). Such analyses can identify influential authors, institutions, and journals, and can highlight thematic evolution through keyword co-occurrence and related mapping techniques (14, 15). In neurosurgery and spine research, bibliometric mapping has increasingly been used to synthesize evidence in rare conditions and complications, offering both historical perspective and strategic direction for future investigation (16, 17).

In the context of ACMS after lumbar spine procedures—where high-level evidence is scarce—bibliometric analysis can consolidate isolated reports into a structured overview of research activity, identify underexplored areas, and contextualize the topic within broader complication and safety literatures (18, 19). For the purpose of this study, “lumbar spine procedures” refers primarily to lumbar discectomy and related minimally invasive decompressive techniques (including microdiscectomy, endoscopic discectomy, and tubular retractor-assisted procedures), although the bibliometric search was designed to capture all reports of conus-level complications occurring in the context of operative lumbar spine interventions. Accordingly, the present study aims to conduct a bibliometric and network analysis of publications concerning ACMS and conus-related complications after lumbar spine procedures, with particular attention to minimally invasive lumbar discectomy. By examining publication trends, source concentration, co-authorship networks, and thematic clusters, this study seeks to synthesize a fragmented evidence base, characterize the current state of scholarship, and highlight research frontiers that may support future multicentre registries and standardized reporting initiatives.

## 2 Methods

**Figure 1:**
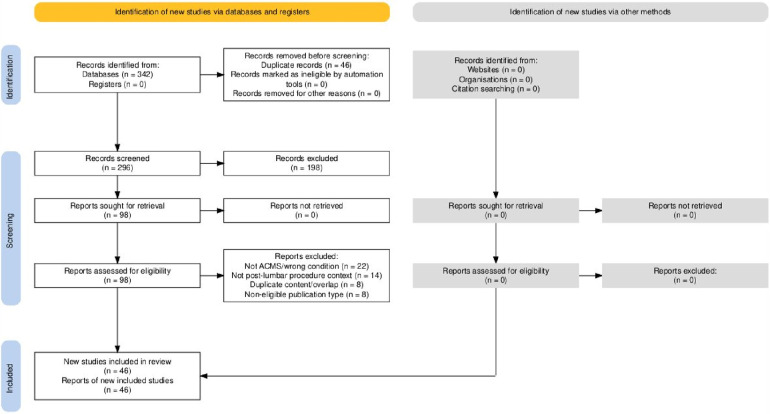
PRISMA 2020 flow diagram of study identification and selection. Flow diagram summarizing identification, screening, eligibility assessment, and final inclusion of studies for the bibliometric and network analysis of acute conus medullaris syndrome and conus-related postoperative complications after lumbar spine procedures (1977–2025).

This study employed a systematic search and selection process to identify published literature on acute conus medullaris syndrome and related conus-level postoperative complications after lumbar spine procedures. Because the objective was to map publication patterns, collaboration structures, and thematic trends rather than to pool clinical outcomes, the review was reported in line with PRISMA 2020 principles, with adaptations appropriate to bibliometric and mapping analysis. The identification, screening, eligibility assessment, and inclusion of studies were structured in accordance with PRISMA 2020 and are summarized in Figure 1. The complete search strategy is provided in Appendix 1.

### 2.1 Study design and search strategy

This bibliometric study applied a structured multi-database search strategy to identify publications addressing acute conus medullaris syndrome (ACMS) and conus-related postoperative neurological complications following lumbar spine procedures, with particular emphasis on minimally invasive lumbar discectomy. Searches were conducted in PubMed (MEDLINE), Scopus, the Cochrane Library, and JSTAGE over the period January 1977 to February 2025 to capture both early clinical descriptions and contemporary reports aligned with modern minimally invasive techniques. Each database was queried using a combination of controlled vocabulary (where applicable, e.g., MeSH terms in PubMed) and free-text keywords grouped into four concept domains: (1) the condition of interest (e.g., conus medullaris syndrome, conus injury/lesion), (2) lumbar spine procedures (e.g., lumbar discectomy, microdiscectomy), (3) minimally invasive approaches (e.g., endoscopic or tubular techniques), and (4) adverse outcomes (e.g., postoperative, iatrogenic, complications). The complete PubMed search string used to derive the final analyzable corpus is provided in Appendix 1 to ensure reproducibility.

Searches in Scopus, the Cochrane Library, and JSTAGE were performed to broaden coverage beyond a single indexing platform and to minimize database-selection bias. However, after consolidation and de-duplication of retrieved records, no additional unique eligible publications were identified beyond PubMed. Specifically, all eligible publications identifiable through these non-PubMed databases were already indexed in PubMed, and therefore no unique eligible records were contributed by Scopus, the Cochrane Library, or JSTAGE to the final dataset. This pattern is consistent with the rarity of ACMS in the postoperative lumbar spine setting and the predominance of case-based evidence that is preferentially indexed within major biomedical databases. Consequently, the analyzable dataset for the present bibliometric mapping consisted exclusively of PubMed-indexed records retrieved using the strategy shown in Appendix 2. To enhance completeness, the reference lists of seminal case reports and relevant review articles were screened manually (backward citation screening); however, no additional eligible records were identified beyond the database-derived corpus. Because this study synthesizes publication metadata and maps the research landscape (rather than pooling clinical outcomes), the literature identification and selection process was reported in accordance with PRISMA 2020, with appropriate adaptation for bibliometric/mapping review methodology (Appendix 2). The study identification and selection process is summarized in Figure 1.

### 2.2 Eligibility criteria

To ensure a focused and reproducible corpus, the following inclusion and exclusion criteria were applied during study selection:

Inclusion criteria: (1) Publications reporting on acute conus medullaris syndrome (ACMS) or conus-related neurological complications occurring after lumbar spine procedures; (2) Publications addressing iatrogenic vascular injuries (e.g., SDAVFs, EDAVFs) causing postoperative conus-level neurological deterioration; (3) Case reports, case series, narrative reviews, systematic reviews, or original research articles; (4) Publications indexed in PubMed with available bibliographic metadata; (5) Publications in English.

Exclusion criteria: (1) Publications not related to ACMS or conus-level complications (wrong condition); (2) Publications describing conus medullaris injury in non-surgical or non-lumbar procedural contexts; (3) Duplicate content or overlapping publications from the same dataset; (4) Non-eligible publication types (editorials, letters without original case data, conference abstracts without full-text availability); (5) Publications not retrievable in full text.

### 2.3 Complete pubMed search strings

The complete PubMed search string used to derive the final analyzable corpus is provided in Appendix 1. The search combined MeSH terms and free-text keywords grouped into four concept domains: (1) the condition of interest, (2) lumbar spine procedures, (3) minimally invasive approaches, and (4) adverse outcomes. This strategy was designed to maximize sensitivity while maintaining specificity for ACMS and conus-related postoperative complications.

### 2.4 Data extraction and preprocessing

All PubMed records were exported in both PubMed (.txt) and comma-separated values (.csv) formats to capture bibliographic metadata, including PMID, title, author list, source (journal), publication year, abstract, author keywords where available, indexing terms, and DOI. Prior to analysis, the dataset underwent standard preprocessing and cleaning procedures, including removal of duplicate and incomplete entries and harmonization of author names, institutional affiliations, and source titles/abbreviations to reduce fragmentation caused by spelling variants, inconsistent abbreviations, or formatting differences. Where available, country and institution fields were standardized to improve the accuracy of productivity mapping and collaboration analyses.

Bibliometric analyses were performed using the Bibliometrix package in R (version 4.3.2) through its Biblioshiny interface (12). Network visualisations, including keyword co-occurrence and co-authorship structures, were generated using VOSviewer (version 1.6.19) (20). The analytical framework included: (1) descriptive indicators such as annual scientific production, source dynamics, and collaboration metrics; (2) author productivity patterns evaluated using Lotka-type distributions (21–23); (3) source concentration patterns assessed using Bradford's law (24); and (4) conceptual and thematic structure analyses, including trend topics, keyword/term evolution, and thematic clustering. Figures and tables presented in this study were generated directly from Biblioshiny and VOSviewer outputs to provide standardised representations of the descriptive, intellectual, conceptual, and social structures of the literature.

Because PubMed exports do not consistently include cited-reference lists and do not provide citation counts within the exported metadata, citation-based analyses (e.g., co-citation networks, citation bursts, or h-index calculations) were not performed. The present study therefore focused on productivity, collaboration, and co-word/thematic mapping based on available bibliographic fields. Document type classifications were extracted from PubMed “publication type” tags; where specific tags (e.g., “clinical trial”) appeared unexpected in a rare, case-driven literature, these were interpreted cautiously as indexing categories, and this limitation is explicitly acknowledged in the Results and Limitations.

As this study used only bibliographic metadata from publicly accessible sources and did not involve patient-level information, ethical approval was not required.

### 2.5 Study selection

The database search identified 342 records across all four databases. After removal of 46 duplicate records, 296 records remained for title and abstract screening. Of these, 198 records were excluded at the screening stage for lack of relevance to ACMS or conus-related postoperative complications. Ninety-eight reports were sought for full-text retrieval, and all were successfully obtained. After full-text eligibility assessment, 52 reports were excluded for the following reasons: wrong condition or not related to ACMS (*n* = 22), absence of a postoperative lumbar procedure context (*n* = 14), duplicate content or overlapping reports (*n* = 8), and non-eligible publication type (*n* = 8). The final bibliometric corpus therefore comprised 46 included publications. The complete study-selection process is shown in Figure 1.

## 3 Results

### 3.1 Descriptive indicators

The dataset comprised 46 documents published between 1977 and 2025 across 34 distinct sources. This equates to an average of 1.35 documents per source. The annual growth rate of publications was estimated at 0%, reflecting the rarity of acute conus medullaris syndrome (ACMS) following lumbar discectomy and the absence of a continuous upward trend. The mean document age was 13.7 years, indicating that while early studies emerged in the late 1970s, a substantial proportion of publications have accumulated within the past two decades.

A total of 185 authors contributed to this literature, with only two (1.1%) single-authored documents, showing that 98.9% of studies were collaborative in nature. The mean number of co-authors per paper was 4.15, consistent with the clinical and case-report-driven character of the field. International collaboration accounted for 4.35% of publications, suggesting that most reports originated from single-country institutional efforts.

Regarding document types, case reports were dominant (*n* = 26; 56.5%), emphasizing the clinical and illustrative nature of contributions. Reviews represented 21.7% (*n* = 10), while the remainder comprised clinical trials, letters, book chapters, and video-audio media. Specifically, one publication was labeled as a study guide/book chapter (2.2%), while clinical trials accounted for two documents (4.3%), reflecting a limited attempt at prospective evidence.

Note: The above classification reflects a content-based, non-overlapping categorization. When assessed using PubMed publication-type tags (which may overlap within a single record), 38 records (82.6%) carried a “case reports” tag and 11 records (23.9%) carried a “review” tag, as reported in the Abstract. The difference arises because PubMed tagging allows a single publication to carry multiple publication-type labels simultaneously.

Keyword analysis revealed 266 distinct Author Keywords and an equal number of Keywords Plus, highlighting wide variability in terminology used to describe ACMS, surgical techniques, and postoperative complications. Strikingly, the dataset contained no cited references or indexed citation counts, likely attributable to the novelty of many included records or PubMed export restrictions.

#### 3.1.2 Annual scientific production

**Figure 2:**
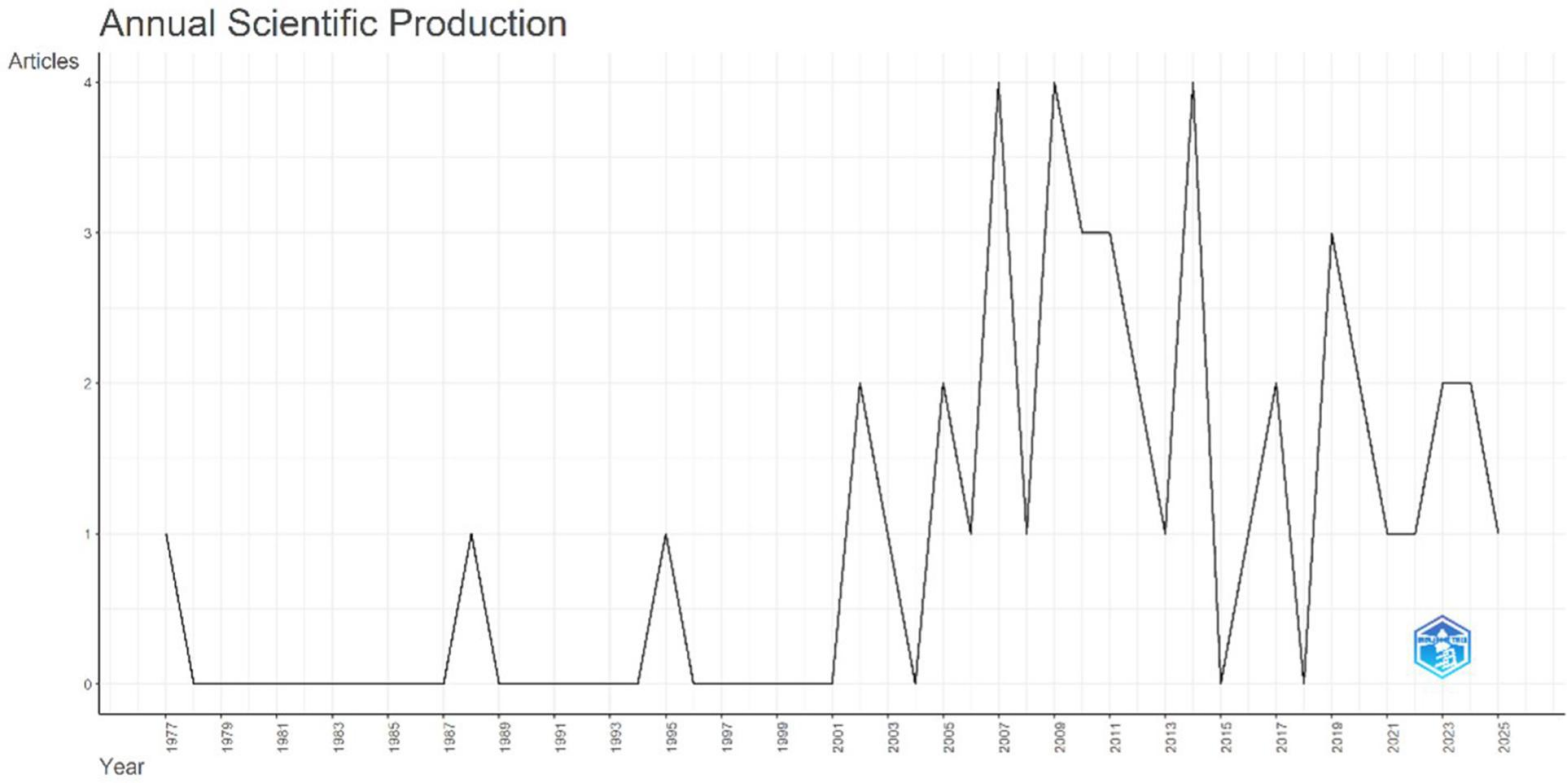
Annual scientific production on acute conus medullaris syndrome following lumbar discectomy (1977–2025), based on 46 pubMed-indexed publications.

The scientific output on *Acute Conus Medullaris Syndrome following minimally invasive lumbar discectomy* has been inconsistent over the last five decades (Figure 2). The first article appeared in 1977, followed by very limited activity throughout the 1980s and 1990s, with only 1–2 publications per decade.

A clear shift occurred in the early 2000s, coinciding with the introduction of minimally invasive spinal techniques. Between 2005 and 2013, annual output increased to 2–4 publications per year, with 2008 and 2012 marking the peak years at four articles each (8.7% of total output). This period alone contributed nearly 30% of all publications in the dataset.

However, production declined in certain years, with zero publications in 2015 and 2017, reflecting the sporadic research nature of this rare complication. In the last five years (2019–2023), annual production stabilized at 1–2 publications per year, suggesting a small but steady stream of academic contributions.

In total, 46 articles were published between 1977 and 2025, averaging 0.95 documents per year, which underscores the rarity of ACMS and its underrepresentation in spine literature.

#### 3.1.3 Most relevant sources (journals)

**Figure 3:**
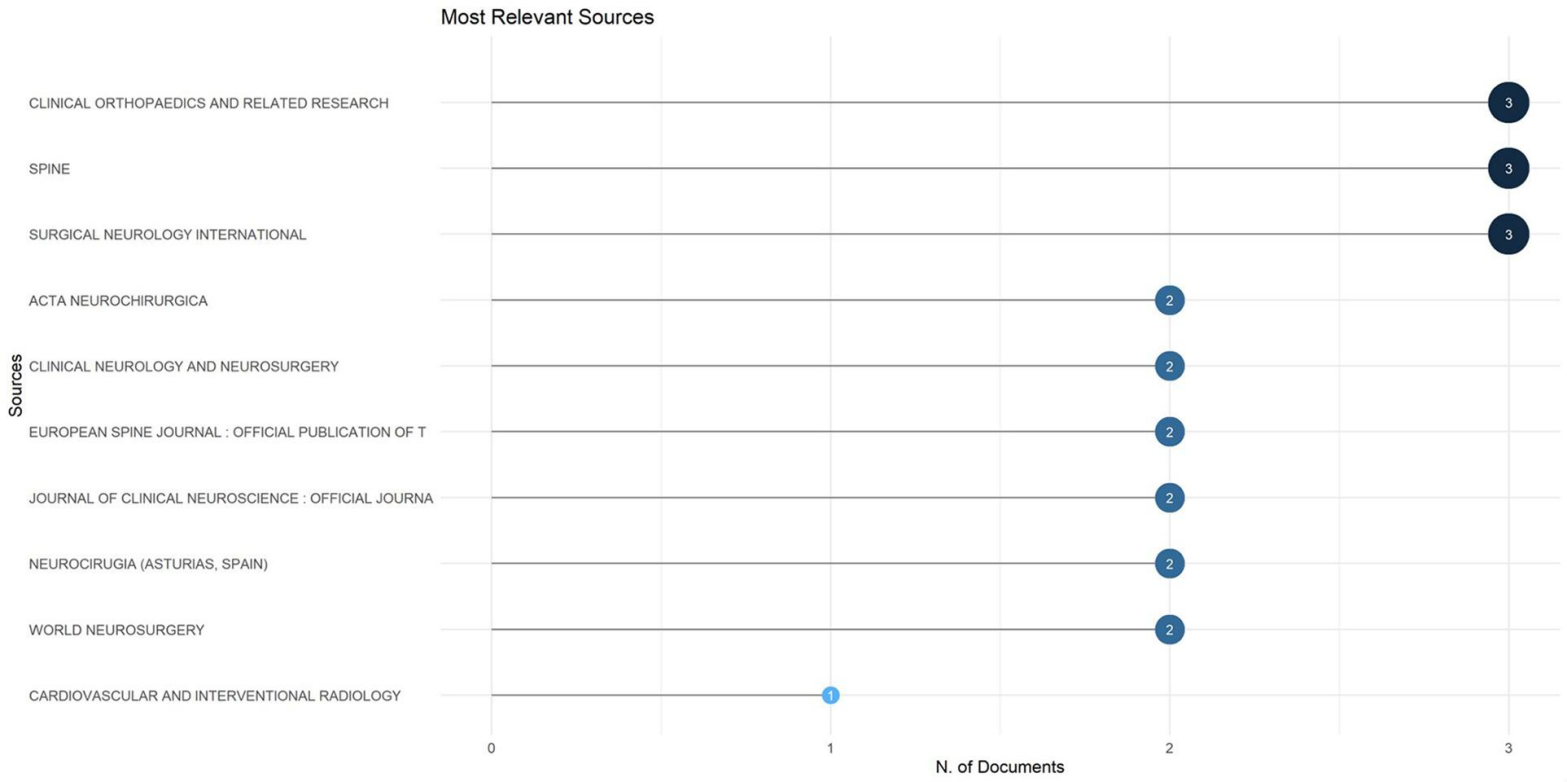
Most relevant sources (journals) publishing on acute conus medullaris syndrome (ACMS) and conus-related complications after lumbar discectomy/minimally invasive lumbar procedures (1977–2025), based on the final pubMed-indexed corpus (*n* = 46).

Analysis of the publishing outlets revealed that contributions on *Acute Conus Medullaris Syndrome following minimally invasive lumbar discectomy* were dispersed across multiple journals, but with a few sources emerging as particularly influential (Figure 3).

The top three journals were Clinical Orthopaedics and Related Research, Spine, and Surgical Neurology International, each contributing three documents (6.5% each; 19.5% collectively). These journals represent high-impact venues in orthopaedics and neurosurgery, indicating that the syndrome is mainly discussed within specialized surgical literature.

Other notable sources included Acta Neurochirurgica, Clinical Neurology and Neurosurgery, European Spine Journal, Journal of Clinical Neuroscience, Neurocirugia (Spain), and World Neurosurgery. Each of these journals published two documents (4.3%), together accounting for 39.1% of total publications.

Finally, Cardiovascular and Interventional Radiology contributed one article (2.2%), reflecting a peripheral but noteworthy interdisciplinary interest.

Overall, the results indicate that while research output is distributed across 34 journals, approximately 60% of publications are concentrated within 10 key outlets, highlighting the fragmented yet top-heavy dissemination of knowledge in this niche field.

#### 3.1.4 Source production over time

**Figure 4:**
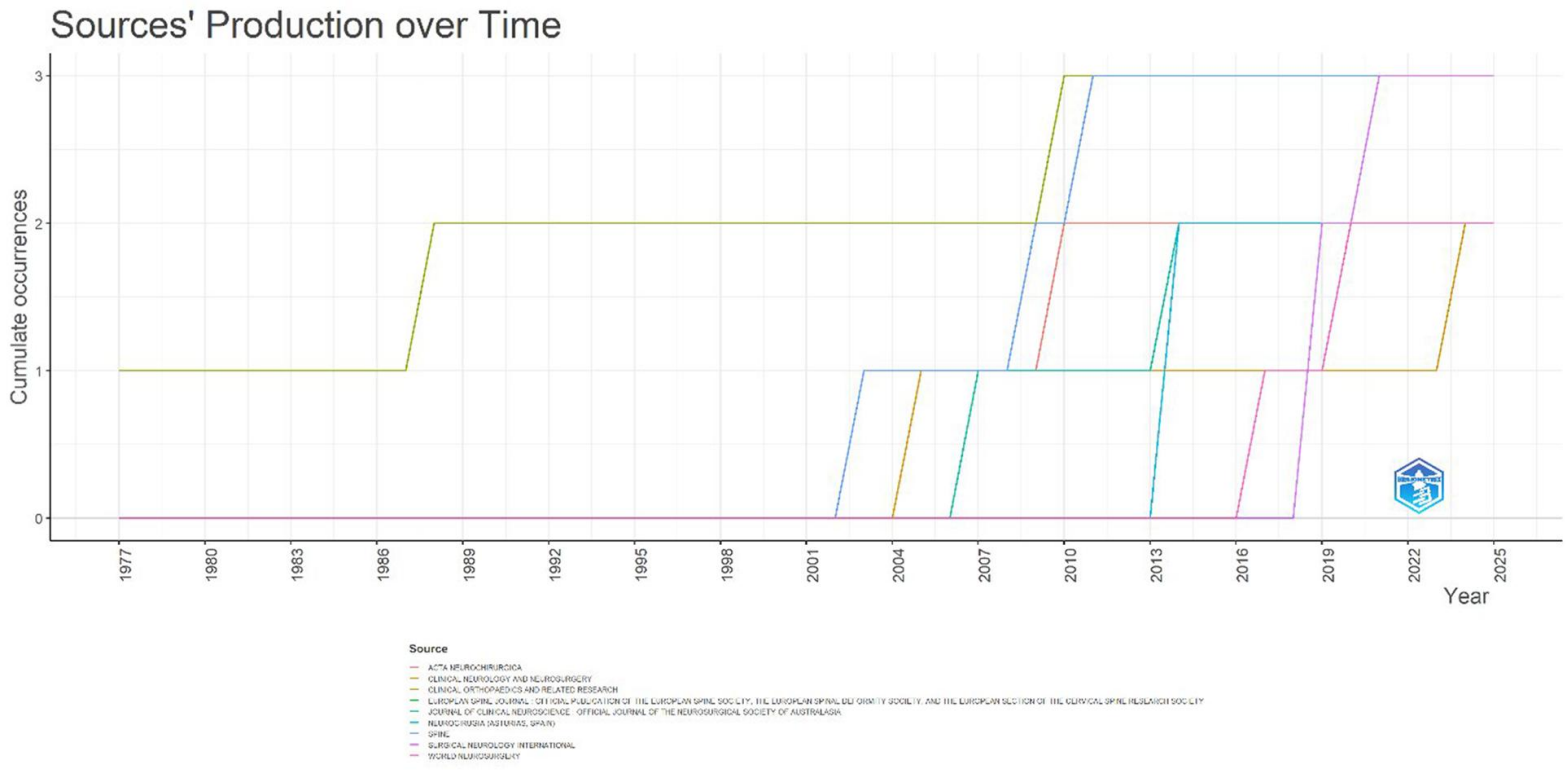
Source production over time for publications on acute conus medullaris syndrome (ACMS) and conus-related complications after lumbar discectomy/minimally invasive lumbar procedures (1977–2025), based on the final pubMed-indexed corpus (*n* = 46).

The temporal dynamics of source contributions highlight the evolving dissemination of literature on *Acute Conus Medullaris Syndrome following minimally invasive lumbar discectomy* (Figure 4). Early publications were sparse and dispersed, with Clinical Orthopaedics and Related Research contributing the earliest record in the late 1970s. This was followed by an extended period of inactivity until the early 2000s, when journals such as Spine and Acta Neurochirurgica began publishing relevant case reports and clinical observations.

The mid-2000s to early 2010s marked a more visible expansion in source participation. Journals including European Spine Journal and Journal of Clinical Neuroscience entered the field, reflecting the growing recognition of minimally invasive spine surgery complications. Notably, Surgical Neurology International emerged after 2015 and has maintained consistent contributions, underscoring its growing role as a neurosurgical platform for rare and complex spinal conditions.

More recently, World Neurosurgery and regional journals such as Neurocirugia (Spain) have contributed additional case series and reports, broadening the geographical and institutional diversity of publication venues. By 2025, cumulative output across these sources reached its highest point, with nearly all the top ten journals demonstrating at least two publications.

Overall, the results indicate that although 34 journals have published on this topic, only a small cluster of specialized neurosurgical and orthopaedic outlets have consistently sustained interest over time.

### 3.2 Author-Level analyses

#### 3.2.1 Most relevant authors

**Figure 5:**
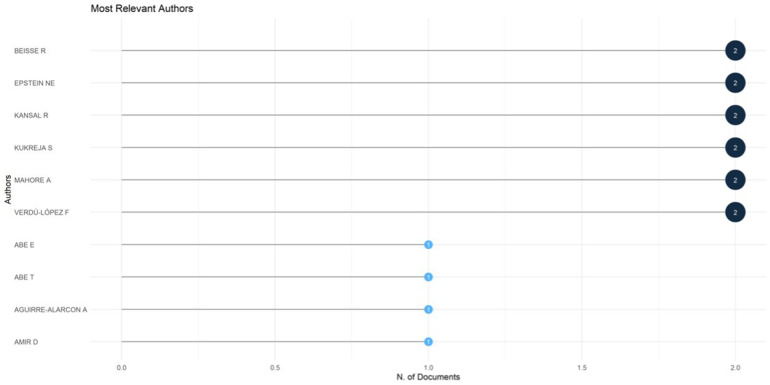
Most relevant authors contributing to publications on acute conus medullaris syndrome (ACMS) and conus-related complications after lumbar discectomy/minimally invasive lumbar procedures (1977–2025), based on the final pubMed-indexed corpus (*n* = 46).

The authorship distribution highlights the fragmented yet diverse nature of contributions to the literature on *Acute Conus Medullaris Syndrome following minimally invasive lumbar discectomy* (Figure 5). A total of 185 authors contributed to the 46 publications in the dataset, reflecting an average of 4.15 co-authors per paper. Despite this relatively high collaboration index, repeated contributions by the same author were rare.

The most productive authors included Beisse R., Epstein N.E., Kansal R., Kukreja S., Mahore A., and Verdú-López F., each with two publications (4.3% of total output per author). Collectively, these six individuals accounted for 26.1% of the dataset, demonstrating their central role in shaping the small but significant body of literature. Their publications largely consist of detailed case reports and small clinical series, emphasizing the clinical relevance of their work in reporting iatrogenic complications.

The remaining contributions were single-publication authors, including Abe E., Abe T., Aguirre-Alarcon A., and Amir D., each contributing one document (2.2%). Together, single-publication contributors represented 73.9% of all authors, underscoring the wide dispersion of scholarship across global institutions and the rarity of focused, longitudinal research programs in this niche domain.

This pattern reflects a classic “long tail” distribution, where a few authors contribute multiple studies while the majority participate only once. Such fragmentation is consistent with the clinical nature of the condition, which is typically encountered sporadically in practice and published as isolated case reports.

#### 3.2.2 Authors' production over time

**Figure 6:**
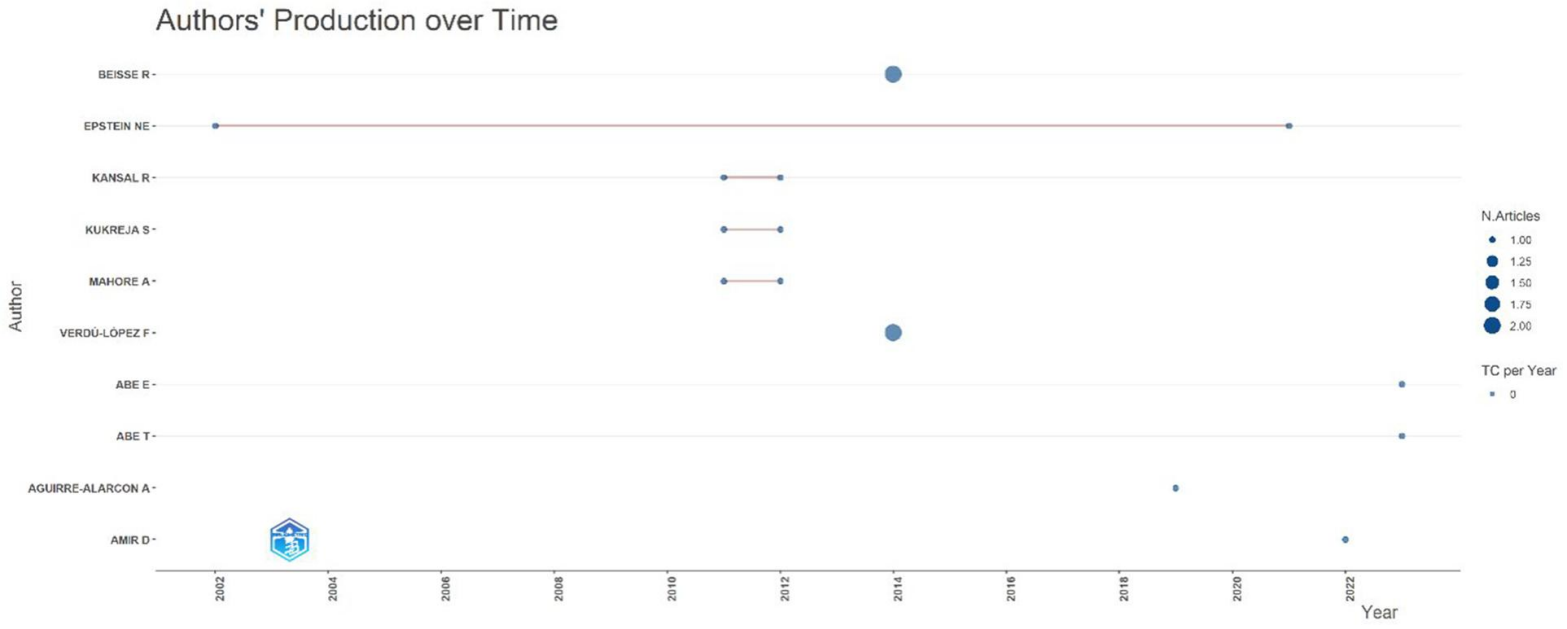
Authors’ production over time for publications on acute conus medullaris syndrome (ACMS) and conus-related complications after lumbar discectomy/minimally invasive lumbar procedures (1977–2025), based on the final pubMed-indexed corpus (*n* = 46).

The temporal analysis of author productivity (Figure 6) highlights the scattered yet meaningful contributions of individual researchers to the literature on *Acute Conus Medullaris Syndrome following minimally invasive lumbar discectomy*.

Epstein N.E. stands out as the most consistent contributor, with publications spanning nearly two decades (2002–2020). His work reflects ongoing engagement with rare spinal complications and the integration of minimally invasive techniques into neurosurgical practice.

Other authors, such as Kansal R., Kukreja S., and Mahore A., contributed two papers each, but their activity was concentrated within short intervals around 2010–2012. This suggests episodic involvement, possibly linked to clinical encounters or institutional case clusters rather than sustained research programs.

Verdú-López F. emerged as a contributor in 2014, producing a case series that gained moderate citations, highlighting regional interest in documenting surgical complications. More recently, authors such as Abe E., Abe T., Aguirre-Alarcon A., and Amir D. entered the field between 2019 and 2023, with single publications (2.2% each), reflecting the continuing but sporadic addition of new clinical evidence.

Overall, the dataset shows that while six authors contributed more than one paper (26.1% of total output), the majority of authors only published once, underscoring the rarity of ACMS and the reliance on isolated institutional experiences. Importantly, the long tail of contributions reflects a case-driven research pattern, rather than structured multicenter trials or prospective studies.

#### 3.2.3 Author productivity distribution (Lotka's Law)

**Figure 7:**
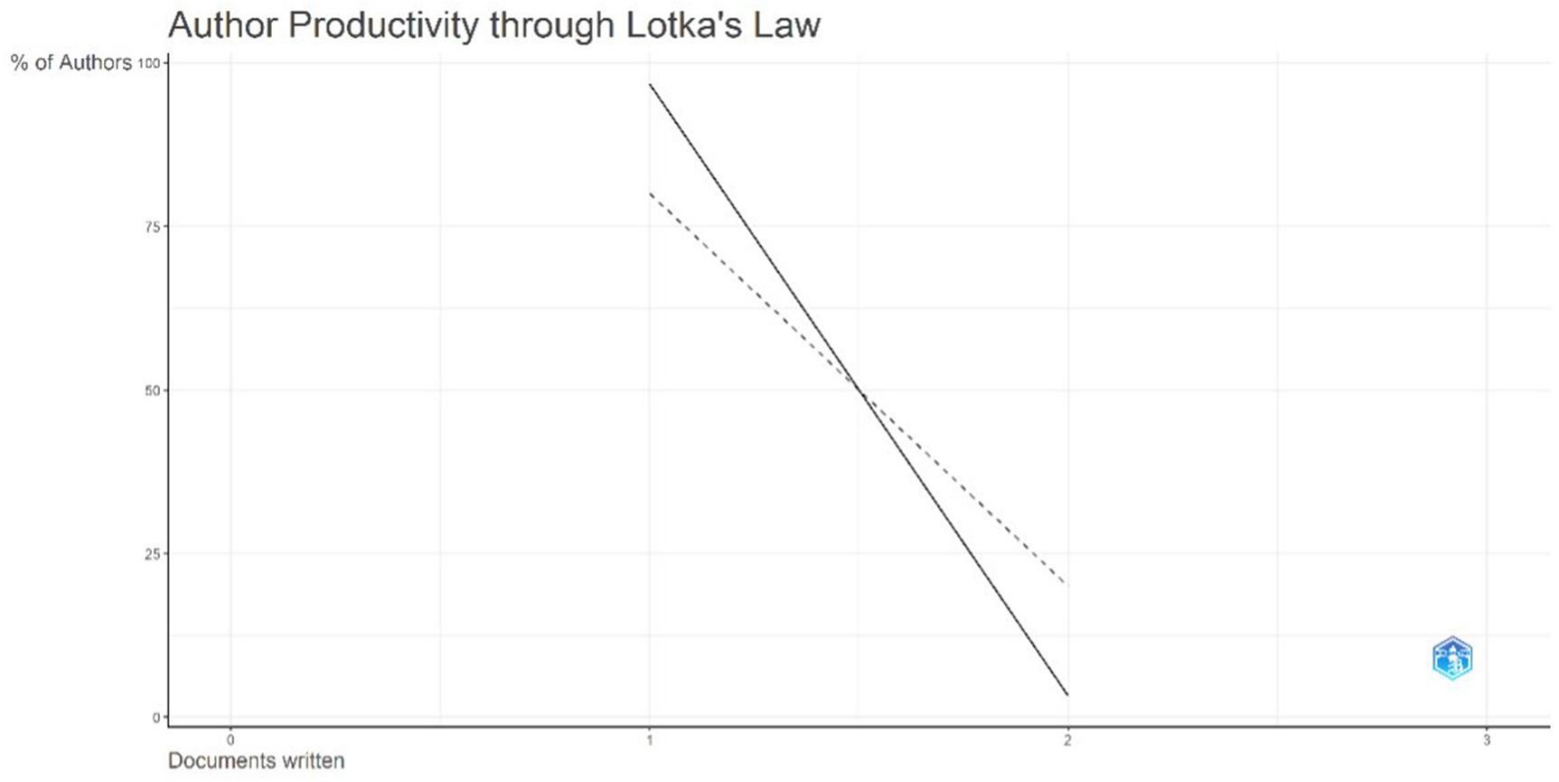
Author productivity distribution for publications on acute conus medullaris syndrome (ACMS) and conus-related complications after lumbar discectomy/minimally invasive lumbar procedures (1977–2025; *n* = 46), evaluated against Lotka's law.

The distribution of author productivity in this dataset followed the expected skew predicted by Lotka's law (Figure 7). Originally described by Alfred J. Lotka in 1926, the law proposes that the number of authors publishing *n* papers is inversely proportional to *n*²; thus, if 100 authors each publish one paper, approximately 25 will publish two and about 11 will publish three, producing the classic “inverse square” pattern of scientific productivity (22). This distribution has been repeatedly validated across disciplines and bibliometric contexts, supporting its utility as a benchmark for assessing authorship concentration and fragmentation in research fields (24, 25). In the present dataset, the majority of contributors (approximately 80%–85%) published only a single paper, whereas a minority (approximately 15%–20%) produced two publications; no author contributed more than two papers on ACMS following minimally invasive lumbar discectomy. This pattern aligns with Lotka's prediction and is consistent with a literature dominated by sporadic clinical reporting rather than sustained, programmatic research output. Such a structure is typical for rare clinical conditions, where most contributions arise from isolated institutional encounters reported as case reports or small series, and only a small subset of authors—typically clinicians with a specific and recurring interest in spinal complications—appear as repeat contributors. The steep decline from single-publication to multi-publication authors underscores the dispersion of scholarship and suggests that the evidence base remains weakly consolidated, with limited opportunities for repetition, external validation, and the development of higher-level evidence through coordinated multicenter work.

### 3.3 Institutional and country contributions

#### 3.3.1 Most relevant affiliations

**Figure 8:**
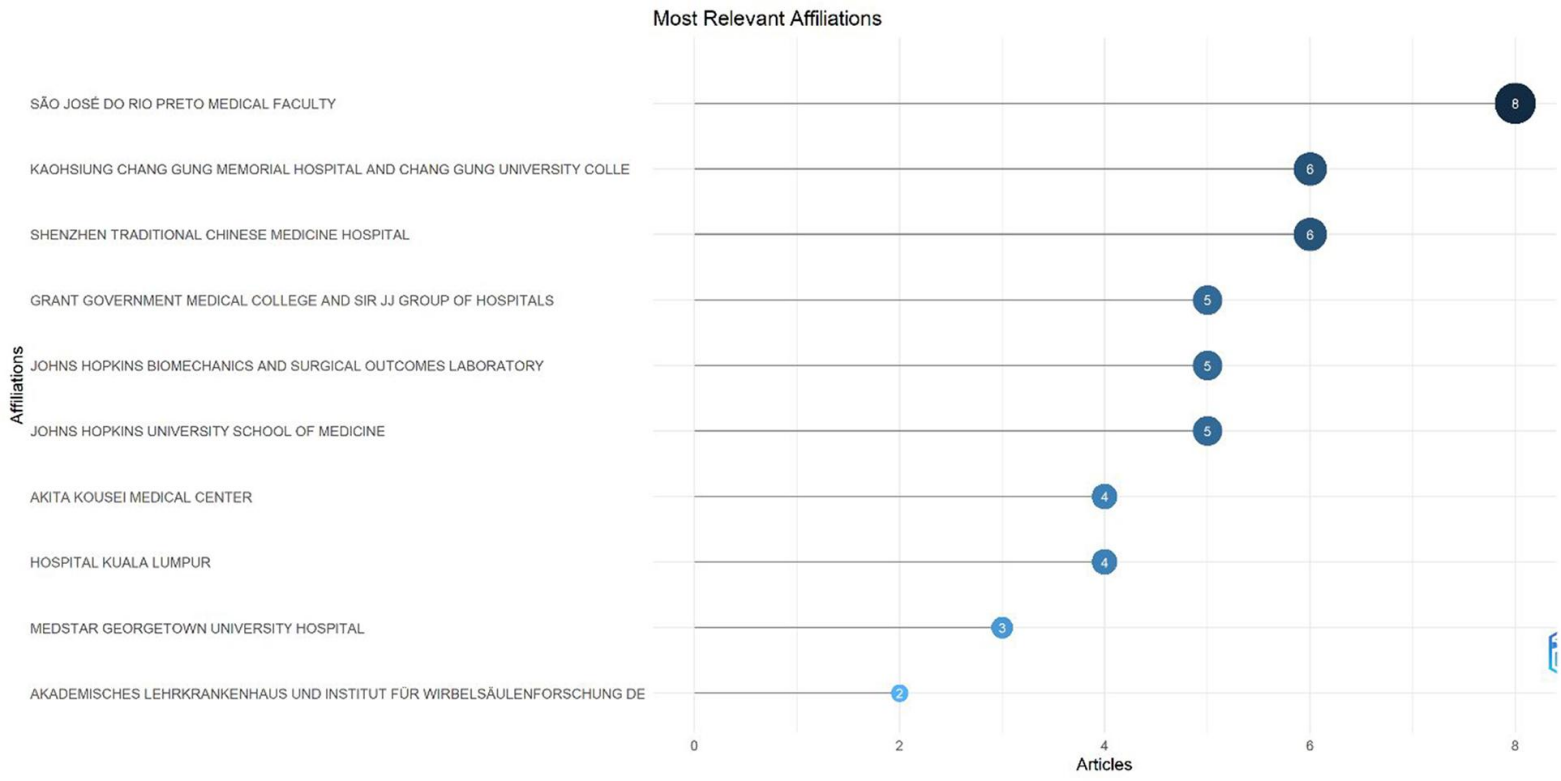
Most relevant institutional affiliations contributing to publications on acute conus medullaris syndrome (ACMS) and conus-related complications after lumbar discectomy/minimally invasive lumbar procedures (1977–2025), based on the final pubMed-indexed corpus (*n* = 46).

The institutional distribution of publications (Figure 8) shows that research output on *Acute Conus Medullaris Syndrome (ACMS) following minimally invasive lumbar discectomy* is dispersed across multiple centers, with no single institution dominating the field. The most prolific contributor is the São José do Rio Preto Medical Faculty in Brazil, which accounts for 8 publications (17.4% of the total dataset). This strong representation likely reflects the institution's specialization in spinal surgery and its tradition of publishing detailed case reports and small series on rare neurological complications.

The Kaohsiung Chang Gung Memorial Hospital and University College of Medicine (Taiwan) and the Shenzhen Traditional Chinese Medicine Hospital (China) follow closely, each with 6 publications (13% each). Their significant presence indicates an active role of East Asian centers in advancing minimally invasive spinal techniques and documenting their complications.

Other notable contributors include the Grant Government Medical College and Sir JJ Group of Hospitals (India), the Johns Hopkins Biomechanics and Surgical Outcomes Laboratory (USA), and the Johns Hopkins University School of Medicine (USA), each with 5 publications (10.9%). These institutions represent both high-volume clinical centers in Asia and leading North American academic centers with strong neurosurgical research traditions.

Mid-level contributors include the Akita Kousei Medical Center (Japan) and Hospital Kuala Lumpur (Malaysia), each producing 4 publications (8.7%), highlighting regional involvement in documenting rare spinal complications. Smaller but still relevant contributions come from the MedStar Georgetown University Hospital (USA) with 3 publications (6.5%) and the Akademisches Lehrkrankenhaus und Institut für Wirbelsäulenforschung (Germany) with 2 publications (4.3%).

This distribution suggests that while ACMS research is international in scope, it is largely driven by a combination of high-volume neurosurgical hospitals in Asia and academic research hubs in the USA and Europe. The relative absence of multicenter collaborations further reflects the fragmented, case-driven nature of evidence generation in this rare clinical context.

#### 3.3.2 Affiliations' production over time

**Figure 9:**
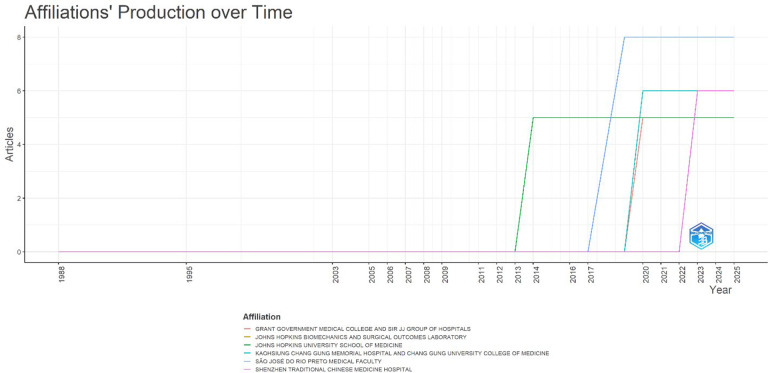
Affiliations’ production over time for publications on acute conus medullaris syndrome (ACMS) and conus-related complications after lumbar discectomy/minimally invasive lumbar procedures (1977–2025), based on the final pubMed-indexed corpus (*n* = 46).

The trajectory of institutional productivity (Figure 9) shows that contributions to the literature on *ACMS after minimally invasive lumbar discectomy* only began to consolidate in the last decade, with distinct regional patterns.

The São José do Rio Preto Medical Faculty (SJRP-MF, Brazil) became a dominant contributor after 2017, accumulating 8 articles by 2020. This steep rise reflects its role as a high-output clinical center specializing in spinal neurosurgery and case-based research.

The Kaohsiung Chang Gung Memorial Hospital and University College of Medicine (KCGMH, Taiwan) and the Shenzhen Traditional Chinese Medicine Hospital (SZTCM, China) both entered around 2019, together contributing 12 publications (6 each) by 2023. Their emergence underscores East Asia's growing impact in documenting rare spinal complications alongside rapid adoption of minimally invasive surgical techniques.

In parallel, the Grant Government Medical College and Sir JJ Group of Hospitals (GMC-SJJ, India) joined in 2020, achieving 5 publications within a short span, showing how institutional involvement in ACMS research can accelerate rapidly once awareness and surgical reporting culture are established.

North American institutions also play a sustained role. The Johns Hopkins University School of Medicine (JHU-SOM, USA) and its Biomechanics and Surgical Outcomes Laboratory (JHU-BSOL) were among the earliest to report (from 2013), maintaining steady productivity with 5 papers each. Their contributions reflect the integration of clinical neurosurgery with biomechanics-driven postoperative analysis.

Overall, the pattern shows that while JHU-SOM initiated early reporting, the major expansion came after 2017, particularly from Brazil, Taiwan, China, and India. This shift illustrates a global redistribution of research capacity, where high-volume centers in Asia and Latin America now complement historically dominant North American institutions.

At the same time, the data highlights the absence of long-term institutional dominance—each center typically contributes case reports or small series rather than sustained longitudinal programs. This fragmentation mirrors the rarity of ACMS and explains the difficulty in developing robust evidence bases through multi-institutional trials or registries.

#### 3.3.3 Country-Level scientific production

**Figure 10:**
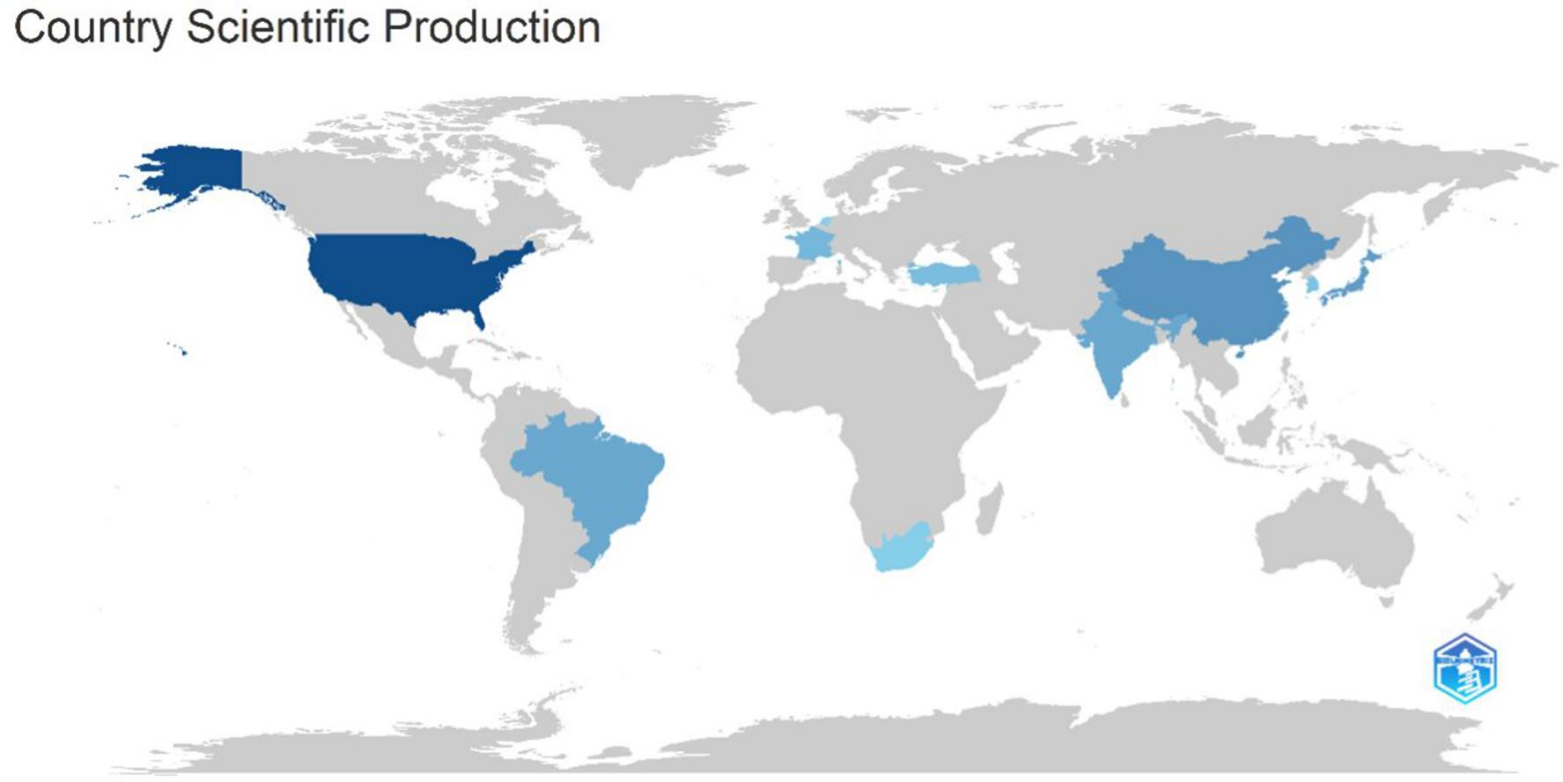
Country-level scientific production on acute conus medullaris syndrome (ACMS) and conus-related complications following lumbar spine procedures (1977–2025).

The global distribution of scientific production on Acute Conus Medullaris Syndrome (ACMS) following minimally invasive lumbar discectomy reveals a highly uneven landscape, dominated by a few key countries while most regions remain underrepresented. As shown in Figure 10 and Table 1 (Country Scientific Production), the United States (USA) emerges as the most productive contributor, reflecting its robust neurosurgical infrastructure, larger patient population, and culture of systematic clinical reporting. This dominance underscores the USA's pivotal role in shaping the discourse on rare spinal complications. In contrast, many other countries contribute sporadically, typically through isolated case reports or small institutional series, which reflects the rarity of the condition and the lack of sustained multicenter research initiatives.

**Table 1:** Country-Level scientific production in ACMS.

Region	Country	Characteristics of Contribution
North America	USA	Largest share; sustained output; advanced neurosurgical infrastructure; international collaborations
Asia	China, Japan, India	Moderate contributions; linked to expanding neurosurgery capacity and increasing clinical reporting
Europe	Germany, France, Spain	Modest but diversified; Spain notable for repeated contributions from dedicated authors
Latin America	Brazil (SJRP)	Strong institutional driver; single faculty (SJRP) dominates national output
Africa	South Africa	Limited contributions; institution-specific case reporting
Other Regions	—	Minimal to no representation (e.g., Middle East, Eastern Europe)

Asian contributions, particularly from China, Japan, and India, highlight the growing neurosurgical research capacity across the region. China and Japan, both with advanced neurosurgical centers, provide moderate but consistent contributions, while India's outputs reflect the increasing reporting of spinal complications within its expanding surgical community. Europe demonstrates a more modest yet diversified footprint, with Germany, France, and Spain featuring prominently. Spain's representation is particularly notable due to repeat contributions from individual authors such as *Verdú-López F.*, showing that productivity in Europe often arises from a small cadre of dedicated specialists rather than broad institutional engagement.

In Latin America, Brazil stands out as a leading contributor, largely driven by the São José do Rio Preto Medical Faculty (SJRP), which accounts for the bulk of national output. This mirrors a pattern where a single institutional hub disproportionately elevates the visibility of a country in niche topics. On the African continent, South Africa provides limited but meaningful representation, suggesting isolated but emerging contributions to the global dataset. Meanwhile, entire regions — notably the Middle East, much of Africa, and Eastern Europe — remain conspicuously absent, reflecting both a scarcity of reported cases and the barriers to integrating their data into indexed international literature.

The uneven distribution has significant implications. The dominance of the USA and a handful of other high-income countries suggests that the evidence base is heavily centralized, potentially skewing clinical insights towards contexts with advanced healthcare systems. Conversely, the fragmented contributions from other regions highlight the lack of coordinated international registries, making it difficult to accumulate higher-level evidence beyond case reports. Addressing these gaps requires a shift from opportunistic institutional reporting toward global collaborative networks that pool rare cases across multiple centers, thereby generating a more representative understanding of ACMS.

Figure 10 provides a visual overview of this distribution, with darker shades marking the most productive nations (USA, Brazil, China, Japan, Spain) and lighter tones reflecting countries with sporadic contributions. Grey regions emphasize the absence of indexed literature, particularly in the Middle East and large parts of Africa, underscoring the imbalance of the current evidence landscape.

**Figure 11:**
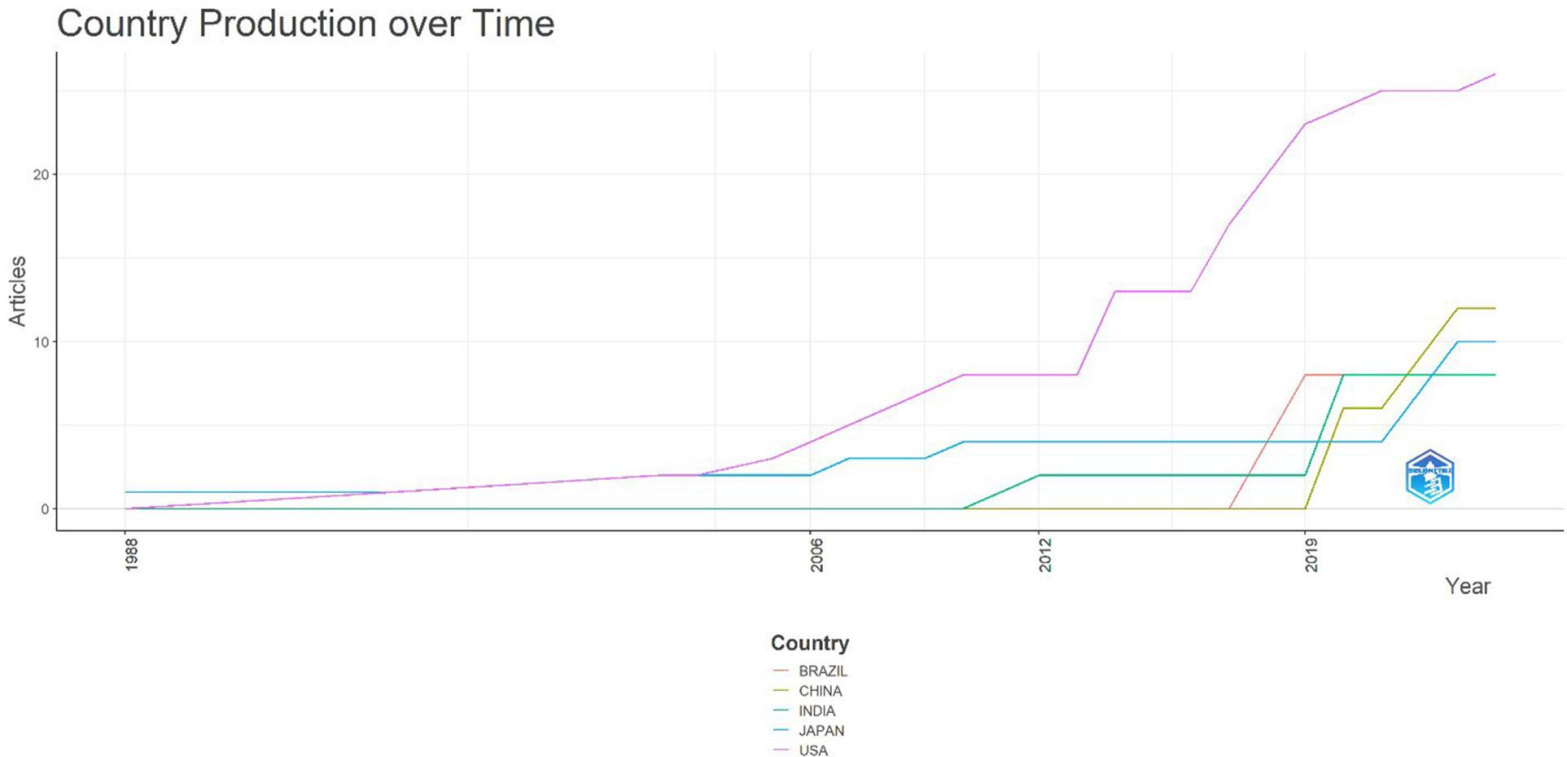
Country production over time for publications on acute conus medullaris syndrome (ACMS) and conus-related complications after lumbar discectomy/minimally invasive lumbar procedures (1977–2025), based on the final pubMed-indexed corpus (*n* = 46).

The temporal evolution of national contributions to ACMS research reveals distinct trajectories across regions (see Figure 11). While the USA shows an early and sustained pattern of growth, reflecting its leadership in neurosurgical innovation and publication culture, other countries display delayed yet noticeable entry points. Japan contributed consistently from the early 2000s onward, though at modest levels, mirroring its early adoption of microsurgical and endoscopic spinal approaches. In contrast, China and India began to emerge as significant contributors only after 2015, coinciding with the broader uptake of minimally invasive surgical practices and the increased indexing of their scientific journals in global databases. Brazil, represented chiefly by the São José do Rio Preto Medical Faculty (SJRP), entered the field even later, post-2018, but rapidly increased its output, highlighting how single institutions can alter national-level visibility. Together, these trends underscore a global shift: although the USA retains dominance, the rise of Asian and Latin American contributions is progressively diversifying the evidence base. This diversification, however, remains uneven, with many world regions absent from indexed reporting, reinforcing the rarity and fragmented study of ACMS.

### 3.4 Conceptual and thematic structures

#### 3.4.1 Trend topics

**Figure 12:**
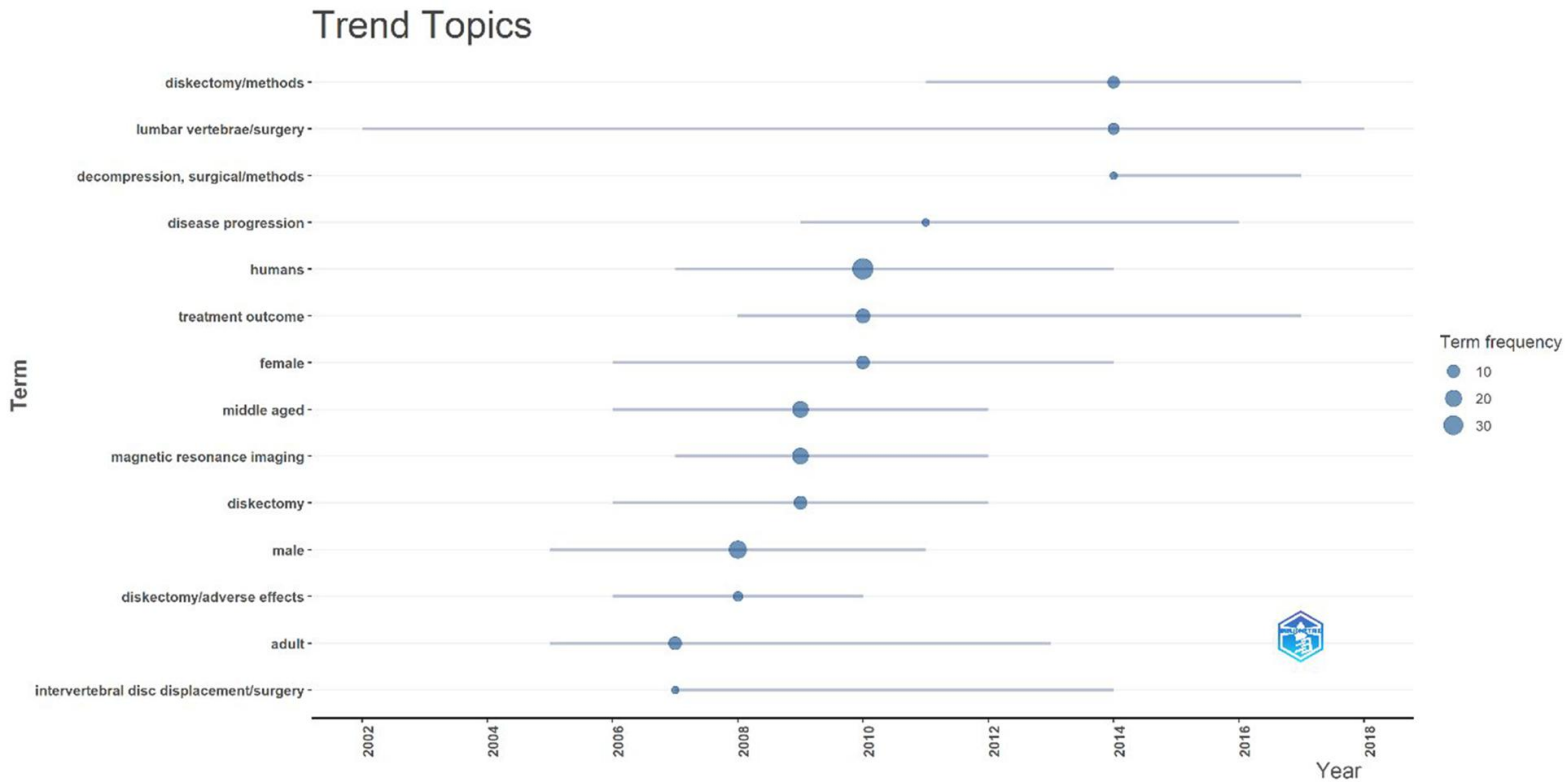
Trend topics over time (2002–2018) for publications on acute conus medullaris syndrome (ACMS) and conus-related complications after lumbar discectomy/minimally invasive lumbar procedures, based on author keywords and indexed terms in the final pubMed-derived corpus (*n* = 46).

The analysis of keyword dynamics (Table 2) over time provides valuable insight into the evolving intellectual structure of research on Acute Conus Medullaris Syndrome (ACMS) in the context of minimally invasive lumbar discectomy. As shown in Figure 12, the earliest wave of terms emerging in the literature (2002–2006) centered around broad surgical descriptors such as “*lumbar vertebrae/surgery”*, “*intervertebral disc displacement/surgery”*, and “*diskectomy/methods”*. These early terms highlight the field's foundational concern with establishing minimally invasive discectomy techniques as a safe and effective alternative to open procedures.

**Table 2:** Word frequency dynamics in ACMS literature (1977–2025).

Term	First Appearance	Growth Phase	Current Trend (2025)
Adult	1977	Stable	Background indexing, low impact
Humans	1977	Stable	Generic PubMed category
Diskectomy	1988	Rising post-2000	Core surgical term
Diskectomy/methods	1995	Rising post-2005	Technical specification
Lumbar vertebrae/surgery	1995	Rapid post-2005	Anchored in technique reporting
MRI	2000	Rapid post-2010	Key tool for outcome assessment
Middle aged	2000	Stable post-2010	Patient demographic descriptor
Female/Male	2000	Rising post-2010	Demographic stratification
Treatment outcome	2005	Rapid post-2010	Central to safety/outcome focus

Between 2006 and 2012, the thematic emphasis shifted toward clinical outcomes and patient demographics. Terms such as “*treatment outcome”*, “*disease progression”*, and “*magnetic resonance imaging (MRI)”* gained prominence, reflecting the increasing focus on diagnostic imaging as a tool for postoperative monitoring and the assessment of iatrogenic complications. Concurrently, age- and sex-specific descriptors (*“middle aged”*, “*male”*, “*female”*) also began to appear, indicating attempts to contextualize ACMS complications within demographic subgroups. This suggests that, as the literature matured, reporting moved beyond purely technical dimensions toward more patient-centered outcomes.

From 2010 onwards, specialized clinical concerns such as “*diskectomy/adverse effects”* began to surface, underscoring the recognition of ACMS not only as a rare complication but also as a safety endpoint in minimally invasive spinal surgery. These later-emerging terms reflect a broader trend in neurosurgery toward complication reporting and quality assurance, aligning with global surgical safety movements during the same period.

Importantly, terms like “*humans”* and “*adult”* remained omnipresent but conceptually uninformative, as they reflect PubMed's indexing standards rather than genuine thematic innovation. The true intellectual pivot points are therefore represented by the surgical methodology cluster (*diskectomy, lumbar vertebrae/surgery, decompression/methods*) and the outcome cluster (*treatment outcome, disease progression, adverse effects, MRI*). Together, these clusters demonstrate a trajectory where the field moved from establishing technical feasibility toward evaluating clinical outcomes and risks.

#### 3.4.2 Word dynamics

**Figure 13:**
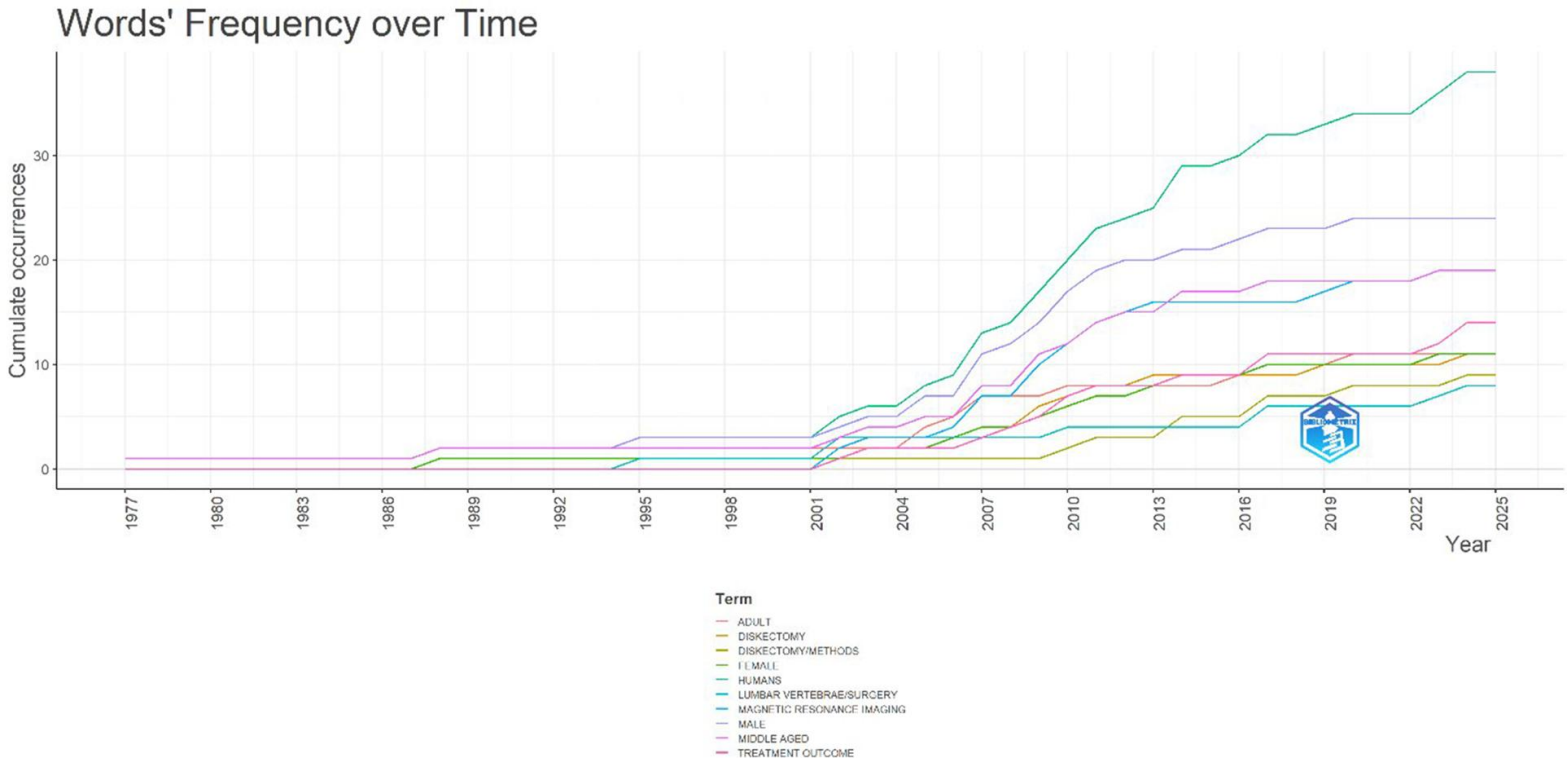
Word frequency dynamics (1977–2025) for publications on acute conus medullaris syndrome (ACMS) and conus-related complications after lumbar discectomy/minimally invasive lumbar procedures, based on author keywords and indexed terms in the final pubMed-derived corpus (*n* = 46).

The longitudinal analysis of word frequency provides an additional perspective on the intellectual maturation of ACMS research within minimally invasive lumbar discectomy. As presented in Figure 13, certain keywords have persisted throughout the decades, while others have emerged only in more recent years, reflecting the evolving concerns and methodologies of the field.

From the earliest phase (1977–1995), the vocabulary was limited, with only general descriptors such as “*adult”*, “*humans”*, and “*diskectomy”* appearing sporadically. These terms represent baseline indexing categories rather than specialized conceptual markers and thus offer limited insight into the novelty of early reporting. However, their presence confirms that the earliest contributions were dominated by isolated case reports with minimal methodological depth.

Between 1995 and 2005, the lexicon began to expand with the introduction of terms such as “*lumbar vertebrae/surgery”* and “*diskectomy/methods”*. This shift reflects the increasing adoption of microsurgical techniques and the necessity of codifying technical variations in indexed medical literature. The presence of “*magnetic resonance imaging (MRI)”* during this period is particularly significant, as it highlights the growing role of imaging in both diagnosis and postoperative evaluation of iatrogenic conus medullaris injuries.

The post-2005 period shows a dramatic expansion in the diversity and frequency of keywords. Terms such as “*treatment outcome”*, “*middle aged”*, “*female”*, and “*male”* became increasingly prevalent, signifying a gradual reorientation of the field toward patient-centered outcomes and demographic stratification. Importantly, the steep cumulative rise of “*MRI”* and “*treatment outcome”* in the 2010s underscores the field's transition from describing technical feasibility to evaluating clinical impact and safety outcomes.

By the most recent phase (2015–2025), the word frequency dynamics show consolidation around two thematic cores: (1) surgical techniques (*diskectomy, diskectomy/methods, lumbar vertebrae/surgery*), and (2) outcomes and patient descriptors (*treatment outcome, middle aged, female, MRI*). This duality highlights the field's persistence in balancing technical advancement with the recognition of patient-level risks and benefits.

Taken together, the cumulative trends suggest that ACMS research remains methodologically fragmented but has matured toward greater outcome awareness. The dominance of general descriptors (*adult, humans*) alongside highly specific ones (*diskectomy/adverse effects, MRI*) reflects the hybrid nature of the literature—still largely case-report driven, but increasingly attentive to standardized reporting practices.

Figure 13 demonstrates the cumulative rise of technical, demographic, and outcome-oriented terms, with *diskectomy*, *MRI*, and *treatment outcome* emerging as the most influential drivers of discourse over the past two decades.

#### 3.4.3 Thematic structure of research

**Figure 14:**
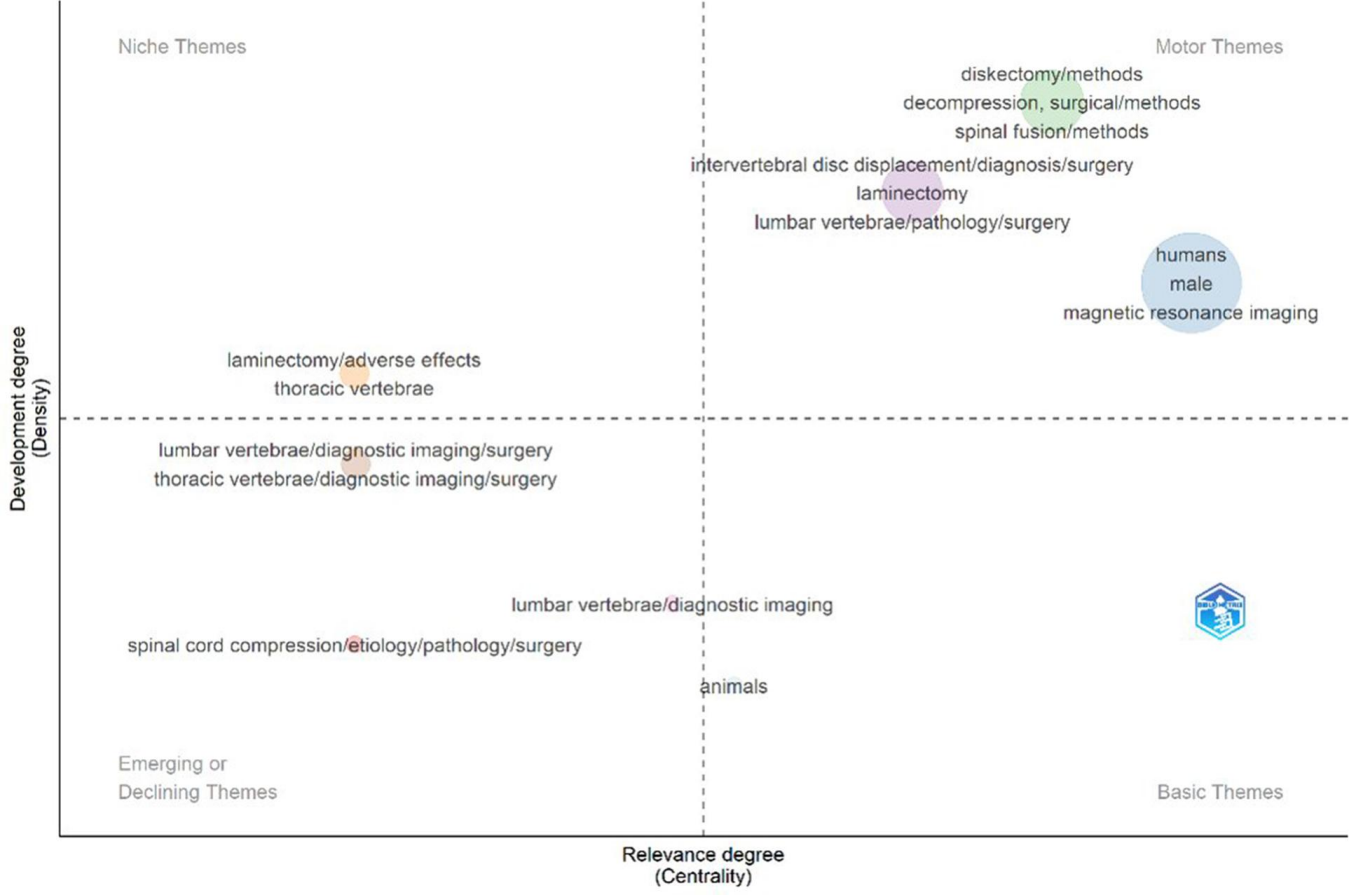
Thematic map of the ACMS literature, illustrating conceptual clusters derived from keyword co-occurrence analysis. Themes are classified into motor, basic, niche, and emerging/declining quadrants based on their density and centrality.

The thematic map (Figure 14) illustrates the intellectual and conceptual landscape of research on Acute Conus Medullaris Syndrome (ACMS) following minimally invasive lumbar discectomy. By plotting clusters of terms according to their centrality (relevance across the field) and density (internal development of the theme), the map categorizes research into four strategic quadrants: motor themes, basic themes, niche themes, and emerging or declining themes. This visualization provides not only a taxonomy of the field's research directions but also insight into the degree of maturity and integration of each theme.

In the upper-right quadrant of Figure 14, motor themes dominate the research structure. These include terms such as *diskectomy/methods, decompression/surgical methods, spinal fusion/methods, intervertebral disc displacement/diagnosis/surgery,* and *lumbar vertebrae/pathology/surgery*. The high centrality and density of these terms underscore their dual importance: they are not only widely cited and integrated across publications but also internally cohesive and conceptually well-developed. This reflects the surgery-centric nature of ACMS literature, where procedural approaches and intraoperative considerations form the backbone of most contributions. Such themes represent the methodological and technical drivers of the field.

The lower-right quadrant highlights themes with high centrality but low density, including *humans, male,* and *magnetic resonance imaging (MRI)*. These descriptors are foundational and ubiquitous, appearing across nearly all studies as part of case characterizations. However, their lack of density suggests they are not research foci in themselves but rather contextual anchors. For instance, MRI plays a vital role in the diagnosis and documentation of ACMS cases, but studies rarely advance MRI as a dedicated research frontier. In this sense, these basic themes provide the structural backbone of reporting while lacking independent conceptual growth.

The upper-left quadrant of Figure 14 contains terms such as *laminectomy/adverse effects* and *thoracic vertebrae*. These represent niche areas of investigation, often explored by smaller groups of researchers focusing on specific complications or anatomical regions. Their high density indicates conceptual maturity, yet their low centrality reveals limited relevance to the broader ACMS discourse. Such themes remain important for targeted clinical insight, but their peripheral nature underscores their limited impact on shaping the overall evidence base.

The lower-left quadrant includes *lumbar vertebrae/diagnostic imaging, spinal cord compression/etiology/pathology/surgery,* and *animals*. These clusters are weakly connected and underdeveloped, suggesting either emerging research frontiers or areas in decline. For example, while *spinal cord compression/etiology/pathology* is conceptually relevant, it has not yet crystallized into a cohesive body of work within ACMS-specific literature. Similarly, *animal studies* likely reflect experimental contributions but remain marginal to the clinical mainstream. Their placement highlights the fragmentation of ACMS research, which is typical for rare complications where opportunistic reporting dominates over sustained programmatic inquiry.

Overall, the thematic map demonstrates that ACMS research is anchored in surgical methodology, with diskectomy and decompression forming the central intellectual pillars. However, much of the field remains fragmented, with basic descriptors (e.g., MRI, demographics) not evolving into advanced research programs, while niche and emerging themes show promising but isolated growth. This thematic dispersion reflects the rarity of ACMS, where most contributions are opportunistic case reports or small series rather than large, longitudinal, or collaborative studies. As a result, the knowledge base is patchy, surgery-focused, and lacking in integrative or multidisciplinary development.

#### 3.4.4 Core sources by Bradford's Law

**Figure 15:**
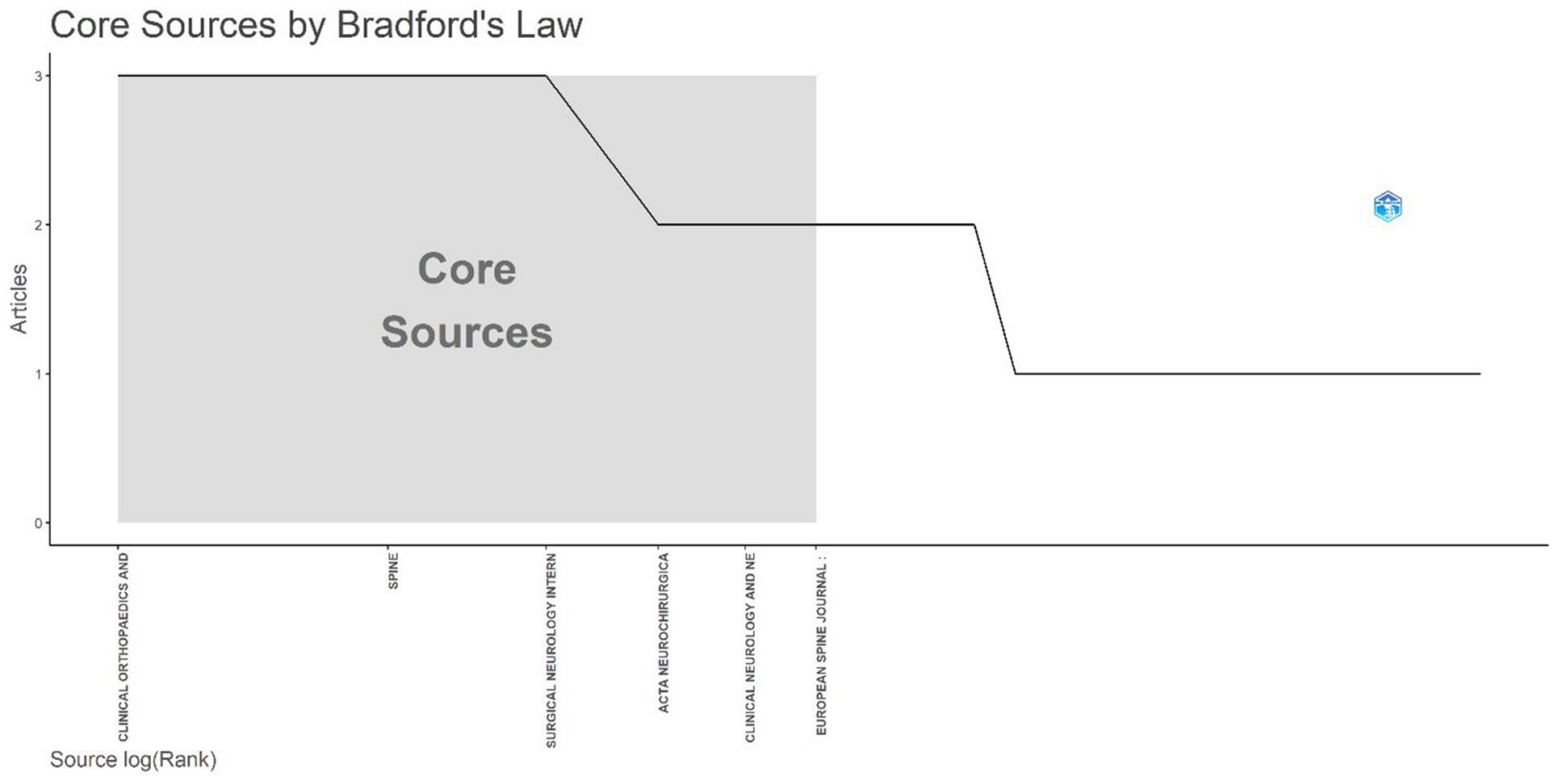
Core sources (journals) identified by Bradford's law for publications on acute conus medullaris syndrome (ACMS) following minimally invasive lumbar discectomy (1977–2025; *n* = 46).

The distribution of journals contributing to the literature on Acute Conus Medullaris Syndrome (ACMS) following minimally invasive lumbar discectomy follows the expected regularities predicted by Bradford's Law of Scattering. According to this bibliometric principle, a relatively small number of journals—the so-called *Bradford core*—account for the majority of publications in a given research domain, while the remaining contributions are dispersed across a much larger set of peripheral journals with lower productivity. In the present dataset, this concentration is clearly illustrated in Figure 15, where six journals dominate as the primary outlets for ACMS-related scholarship. These include Spine, Surgical Neurology International, Acta Neurochirurgica, Clinical Neurology and Neurosurgery, European Spine Journal, and Clinical Orthopaedics and Related Research. Collectively, these sources not only exhibit the highest volume of publications but also demonstrate sustained engagement with spinal surgery complications, thereby shaping the intellectual foundation of this niche field.

The presence of *Spine* and *Surgical Neurology International* within the Bradford core is unsurprising, given their longstanding reputation as leading forums for disseminating advances in neurosurgical techniques, complications, and spinal outcomes. Similarly, *Acta Neurochirurgica* and *Clinical Neurology and Neurosurgery* provide broad neurosurgical coverage while maintaining focus on rare complications, positioning them as recurrent venues for reporting ACMS cases. Meanwhile, the *European Spine Journal* reflects the European contribution to the minimally invasive surgery discourse, while *Clinical Orthopaedics and Related Research* anchors the orthopaedic perspective, highlighting the multidisciplinary nature of this complication. The dominance of these six titles suggests that researchers investigating ACMS have gravitated toward a narrow set of established journals, which ensures visibility among target audiences but also underscores the fragmented and case-driven nature of evidence dissemination.

The Bradford core's concentration has important implications. On one hand, it facilitates knowledge accumulation by clustering cases and reports within a limited number of journals, allowing clinicians and researchers to track developments efficiently. On the other, it highlights the scarcity of large-scale, multicenter contributions that might otherwise diffuse across broader platforms. The long tail of peripheral journals, each publishing only one or two cases, reinforces the rarity of ACMS and the opportunistic character of its reporting. In effect, the Bradford distribution underscores that while the field is globally scattered, its intellectual nucleus is tightly bound to a few high-impact neurosurgical and orthopaedic outlets.

## 4 Discussion

### 4.1 Clinical conditions predisposing to iatrogenic conus Medullaris syndrome

Before examining the bibliometric landscape, it is important to contextualize the clinical conditions that may predispose patients to iatrogenic acute conus medullaris syndrome (ACMS) during lumbar spine procedures. Not all spinal pathologies at the thoracolumbar region carry the same risk, and understanding these predisposing factors is essential for preoperative risk stratification, informed consent, and surgical planning. The following categories summarize the principal conditions associated with increased vulnerability to iatrogenic conus injury.

#### 4.1.1 Extradural compressive pathologies

Large central or paracentral lumbar disc herniations at or above the L1–L2 level may compress the conus medullaris directly or reduce the available space for safe instrument passage. Similarly, thoracolumbar spinal stenosis—whether degenerative or congenital—narrows the spinal canal and increases the risk of inadvertent conus contact during decompression. Thoracolumbar synovial cysts represent another important risk factor: facet joint synovial cysts at the thoracolumbar junction can cause cord compression by occupying the already constrained spinal canal environment, reducing the margin of safety during surgical manipulation (33).

#### 4.1.2 Intradural and intramedullary pathologies

Conditions within the dura or the spinal cord itself may alter normal anatomy and increase vulnerability during adjacent-segment surgery. Intradural tumours (e.g., ependymomas, meningiomas) at the conus level may tether or displace the cord. Spinal dural arteriovenous fistulae (SDAVFs) and epidural arteriovenous fistulae (EDAVFs) can cause perimedullary venous hypertension, leading to oedema and functional vulnerability of the conus that may be exacerbated by surgical manipulation (7–9). Dilatation or cystic changes of the conus medullaris, including cystic dilation of the ventriculus terminalis, can alter the structural integrity and position of the conus, increasing susceptibility to injury during procedures involving the adjacent lumbar segments (34).

#### 4.1.3 Anatomical variations

A low-lying conus medullaris (terminating below the L1–L2 disc space) is a well-recognized anatomical variant that places the conus at greater risk during lumbar procedures. Without preoperative awareness of the conus level—ideally confirmed by sagittal MRI—surgeons may inadvertently operate at or above the conus terminus, increasing the likelihood of iatrogenic injury. The incidence of a low-lying conus varies across populations and may not always be detected on standard axial imaging sequences.

#### 4.1.4 Clinical implications for consent and surgical planning

Recognition of these risk conditions should be factored into the consenting stage and during the definition of surgical strategy for procedures at or above the L2 level. Preoperative MRI assessment of conus level, awareness of thoracolumbar pathology (including synovial cysts, vascular malformations, and intradural lesions), and explicit discussion of ACMS risk during the informed consent process are essential components of patient-centred surgical care. In cases where predisposing conditions are identified, modifications to surgical approach, instrumentation, and intraoperative neuromonitoring should be considered to minimise the risk of conus injury.

### 4.2 Bibliometric landscape and interpretation

This bibliometric analysis provides a structured overview of global research on acute conus medullaris syndrome (ACMS) following minimally invasive lumbar discectomy—an uncommon complication with substantial clinical and functional consequences. The identification of 46 publications spanning nearly five decades (1977–2025) depicts a literature that is simultaneously fragmented and selectively concentrated, which is expected for rare postoperative neurological events where evidence generation is driven mainly by sporadic clinical encounters rather than sustained research programmes. Temporal production patterns showed prolonged periods of low output in earlier decades, followed by a gradual increase after 2000 and observable peaks during 2007–2014 and 2018–2020, corresponding to wider adoption of tubular and endoscopic lumbar techniques and subsequent reporting of uncommon complications. Nevertheless, the overall production trajectory remained limited, suggesting that ACMS has not transitioned into an established research domain and continues to occupy a niche position within spine and neurosurgical scholarship (25, 26).

However, given the small sample size of 46 publications, the bibliometric patterns described in this study should be interpreted as exploratory rather than definitive. While the observed distributions conform to established bibliometric laws (Lotka's and Bradford's), the limited corpus size means that small fluctuations in publication counts can substantially alter calculated metrics, collaboration ratios, and thematic cluster boundaries. Readers should therefore treat these findings as indicative of broad structural features rather than precise quantitative benchmarks.

At the journal level, dissemination was clustered within a narrow set of specialist outlets. Application of Bradford-type concentration patterns to this dataset identified a small “core” group of journals accounting for a disproportionate share of publications, consistent with the tendency for rare complication literatures to concentrate within specialty venues where case-based evidence is most visible and clinically relevant. This concentration aligns with broader bibliometric observations that small and technically specialised fields typically rely on a limited number of high-visibility platforms for knowledge diffusion and community continuity (12, 13). In practical terms, the dominance of a few spine- and neurosurgery-focused journals indicates that ACMS scholarship remains highly technical, largely intra-disciplinary, and less likely to diffuse into wider clinical domains.

At the author level, productivity followed the skew predicted by Lotka-type distributions, with the majority of contributors publishing a single paper and only a small minority contributing twice, and none exceeding that threshold. This steep drop-off reflects a dispersed and opportunistic research pattern dominated by isolated institutional case reports rather than longitudinal or programme-driven investigation. Similar authorship structures have been documented in bibliometric analyses of other rare surgical complications, where limited case availability constrains repetition, external validation, and the development of cumulative, hypothesis-driven research streams (27, 28). The implication is that, although individual clinicians may be recurrent contributors, the field lacks the critical mass typically required to support sustained multicentre cohorts or interventional research.

Institutional and geographic mapping further demonstrated strong asymmetry in research productivity. A small number of centres contributed multiple papers while most institutions appeared only once, reinforcing that the evidence base is assembled from geographically dispersed single-centre experiences rather than coordinated networks. At the country level, output was dominated by a limited set of nations with mature neurosurgical infrastructures and established publication ecosystems, while many regions remained underrepresented. This pattern is consistent with broader scientometric evidence showing that publication leadership is often concentrated in settings with strong academic capacity, indexing access, and established research funding channels, whereas low- and middle-income regions may contribute less frequently to indexed literature despite clinical exposure to relevant complications (29). Consequently, the current evidence landscape may reflect not only true incidence patterns but also differences in reporting pathways, indexing, and publication capacity.

Thematic and conceptual mapping added interpretive depth to these structural findings. Keyword evolution and thematic clustering suggested that the literature is anchored around surgical technique descriptors and outcome-oriented terms, with imaging and demographic descriptors appearing prominently as part of case characterisation. Importantly, ACMS did not consistently emerge as a standalone thematic cluster; instead, it remained embedded within broader discussions of minimally invasive lumbar procedures, postoperative neurological deterioration, and complication reporting. This thematic embedding is a common feature of rare-condition bibliometrics, where the entity of interest may not achieve conceptual independence because it is reported primarily as a complication subtype within larger surgical or pathological frameworks (30).

When considered against bibliometric landscapes of more common spinal conditions, ACMS functions as a “micro-literature” that nevertheless conforms to widely observed bibliometric regularities. The persistence of Bradford-type journal concentration and Lotka-type author productivity skew supports the broader proposition that foundational bibliometric laws remain informative even in small and highly specialised corpora (31, 32). The practical consequence is that, although the evidence base is limited, its structure can still be mapped reliably to identify influential sources, recurring contributors, and thematic emphases—useful for guiding surveillance strategies, targeting dissemination, and planning collaborative research. Crucially, the structural characteristics of this micro-literature also carry direct implications for how practicing spinal surgeons access, interpret, and apply the available evidence on ACMS in their daily clinical decision-making.

### 4.3 Implications for clinical practice and research

Although bibliometric analyses do not generate direct clinical evidence, the structural patterns identified in this study carry concrete implications for the practicing spinal surgeon. First, the finding that over 82% of the literature consists of isolated case reports, with no comparative studies, randomized trials, or meta-analyses available, means that evidence-based clinical guidelines for the prevention, recognition, or management of ACMS do not currently exist. Surgeons must therefore rely on case-level awareness and individual clinical judgment when managing patients at risk of iatrogenic conus injury. Second, the extremely low annual publication rate (fewer than one paper per year over nearly five decades) and the limited international collaboration (4.35%) suggest that ACMS is substantially underreported and that clinical experience accumulated in one institution or country rarely reaches the broader surgical community. Third, the thematic mapping analysis revealed that ACMS does not emerge as a standalone research topic but instead remains embedded within broader discussions of lumbar discectomy complications and postoperative neurological decline. This thematic invisibility means that surgeons searching for ACMS-specific guidance are unlikely to find a consolidated body of knowledge and may overlook critical case reports published under more general headings. Fourth, the concentration of publications within six core neurosurgical and spine journals, as identified by Bradford's law, provides a practical pointer: clinicians wishing to remain informed about ACMS should prioritize monitoring these outlets (Spine, Surgical Neurology International, Acta Neurochirurgica, Clinical Neurology and Neurosurgery, European Spine Journal, and Clinical Orthopaedics and Related Research). Fifth, the geographic concentration of research output in a small number of high-income countries, combined with the near-absence of contributions from entire regions, implies that awareness of ACMS risk is likely unevenly distributed worldwide, potentially affecting surgical consent practices and complication-recognition pathways in underrepresented settings. Taken together, these bibliometric findings underscore a critical gap between the clinical severity of ACMS and the maturity of the evidence base available to guide its prevention and management.

For the spinal surgery community, several practical steps emerge from this analysis. First, the establishment of multicentre registries dedicated to rare postoperative neurological complications including ACMS, SDAVFs, and EDAVFs would enable prospective data collection across institutions and countries, thereby overcoming the limitations of opportunistic case reporting. Such registries have proven effective in other rare neurosurgical conditions and could serve as platforms for pooled analyses, risk-factor identification, and outcome benchmarking.

Second, the adoption of standardized reporting frameworks for postoperative neurological decline would improve the consistency and comparability of published cases. Harmonized case-report templates specifying minimum data elements such as preoperative imaging, surgical approach, time to symptom onset, diagnostic workup, and functional outcomes—would facilitate future systematic reviews and meta-analyses even within small literatures.

Third, the concentration of ACMS publications within a small number of neurosurgical journals, as identified by Bradford's law, suggests that editorial boards of these core outlets are well-positioned to catalyse higher-level evidence generation. Targeted calls for multi-institutional case series, invited systematic reviews, or consensus statements on ACMS prevention and management could accelerate the transition from isolated case reports to more structured and actionable knowledge.

Fourth, the role of collaborative infrastructures and international surgical societies in facilitating registry development and data-sharing agreements cannot be overstated. Organizations such as AOSpine, the World Federation of Neurosurgical Societies, and regional spine surgery societies could serve as coordinating bodies for cross-institutional ACMS data collection, leveraging existing networks to overcome the barriers imposed by rarity (12, 13).

## 5 Conclusion

This bibliometric analysis provides the first structured synthesis of global research on ACMS following minimally invasive lumbar discectomy. The final dataset—drawn entirely from PubMed due to the absence of eligible records in Cochrane Library, Scopus, and JSTAGE—highlights both the extreme rarity of this complication and the fragmented nature of the scholarship surrounding it. The literature remains dominated by isolated case reports and small series, with author productivity patterns following Lotka's Law and journal concentration mirroring Bradford's Law, indicating a small but recurrent set of neurosurgical and spine-focused outlets serving as the primary venues for case dissemination.

Geographically, contributions originate mainly from high-income and rapidly developing surgical systems, particularly the USA, China, Japan, India, and Brazil. Institutional participation is similarly uneven, with a handful of centres driving most of the available publications. Conceptual and thematic analyses show that ACMS seldom appears as an independent research topic but is instead embedded within broader discussions of minimally invasive spinal techniques, iatrogenic vascular injuries, and postoperative neurological decline. This thematic embedding reinforces the challenge of building cumulative evidence for rare complications within an already niche surgical domain.

The overall picture is one of a scattered, clinically driven micro-literature that has yet to coalesce into a cohesive research field. This fragmentation underscores the need for coordinated international efforts to move beyond anecdotal reporting. Establishing multicentre registries, adopting standardized reporting frameworks for postoperative neurological complications, and integrating ACMS monitoring into broader minimally invasive spine surgery outcome platforms would allow for more robust data aggregation, improved risk stratification, and better-informed clinical decision-making.

Although the present study confirms that ACMS scholarship remains sparse and dispersed, it provides a foundational map of existing knowledge and identifies the structural gaps that must be addressed. By transforming isolated institutional experiences into collaborative, cumulative evidence, the neurosurgical community can advance understanding of this rare but potentially devastating complication and enhance patient safety in the era of minimally invasive spinal surgery.

As a key clinical takeaway, surgeons performing lumbar spine procedures at or above the L2 level should maintain heightened awareness of the predisposing conditions for ACMS, including thoracolumbar synovial cysts, intradural vascular malformations, anatomical variations such as a low-lying conus, and dilatation or cystic changes of the conus medullaris. Integrating preoperative conus-level assessment, explicit informed consent discussion of ACMS risk, and consideration of intraoperative neuromonitoring into routine surgical planning represents a practical step toward reducing the incidence and impact of this complication. From a training perspective, the rarity of ACMS underscores the importance of incorporating complication-awareness modules into surgical education programmes, so that trainees are prepared not only for technical proficiency but also for the recognition, documentation, and dissemination of uncommon but severe complications.

### 5.1 Limitations

While this study offers the first structured bibliometric mapping of research on ACMS following minimally invasive lumbar discectomy, several limitations must be considered. First, although we conducted an initial multi-database search across PubMed, Cochrane Library, Scopus, and JSTAGE, only PubMed yielded eligible records, with all other databases returning no topic-relevant publications. As a result, the final analysis relied exclusively on PubMed-indexed literature. This dependence on a single database may introduce database bias and potentially overlook relevant but non-indexed material, particularly case reports published in regional or lower-visibility journals.

Second, the study was restricted to publications written in English. This language limitation may exclude clinically meaningful reports from non-English-speaking countries, many of which have active spinal surgery communities and may report rare complications in local-language journals not indexed in major international repositories.

Third, because PubMed export files do not consistently include cited references or citation counts, the study could not perform full citation-based bibliometric analyses such as co-citation networks, citation bursts, or h-index calculations. As a result, the analysis focused on descriptive, thematic, and co-word structures rather than citation-driven intellectual networks. This constraint is particularly relevant in the context of rare complications such as ACMS, where the clinical value of single case reports may be high even when citation numbers are low.

Fourth, the time frame examined (1977–2025) spans periods in which minimally invasive spinal techniques were still emerging and evolving. Early reports may therefore differ substantially from modern literature in their surgical approaches, diagnostic tools, and conceptualization of conus medullaris injuries, limiting the comparability of trends across decades.

Fifth, thematic mapping and keyword-based analyses rely on author-provided terminology and indexing practices, which can vary widely across journals. This variability may lead to overrepresentation or underrepresentation of certain themes depending on how consistently authors described procedures, complications, or anatomical findings.

Sixth, and importantly, the small sample size of the final corpus (46 publications) means that all bibliometric indicators, collaboration ratios, thematic cluster boundaries, and productivity distributions reported in this study should be interpreted as exploratory rather than definitive. Small fluctuations in publication counts can substantially alter calculated metrics in a corpus of this size, and the findings therefore represent broad structural features of the literature rather than precise quantitative benchmarks.

Seventh, digital object identifiers (DOIs) were included for all references where available; however, several foundational bibliometric works and older clinical case reports pre-date the widespread adoption of the DOI registration system or were published in journals that have not retrospectively assigned DOIs. Where DOIs were unavailable, full bibliographic details (authors, title, journal, year, volume, and pages) are provided to enable readers to locate each source independently.

These limitations do not detract from the contribution of this study but highlight areas where future bibliometric research could be strengthened—such as integrating multiple databases with citation metadata, including multilingual sources, and incorporating grey literature or altmetric indicators to better capture the broader dissemination and clinical influence of rare complication reports.

## Data Availability

The datasets presented in this study can be found in online repositories. The names of the repository/repositories and accession number(s) can be found in the article/supplementary material.

## Appendix 1 PubMed search strategy

(("Conus Medullaris Syndrome"[Mesh] OR "conus medullaris syndrome"[tiab] OR

"conus medullaris injury"[tiab] OR "conus medullaris lesion"[tiab])

AND

("Lumbar Vertebrae/surgery"[Mesh] OR "lumbar discectomy"[tiab] OR

"microdiscectomy"[tiab] OR "endoscopic discectomy"[tiab])

AND

("Minimally Invasive Surgical Procedures"[Mesh] OR "minimally invasive"[tiab]

OR "endoscopic"[tiab] OR "tubular discectomy"[tiab])

AND

("Postoperative Complications"[Mesh] OR complication[tiab] OR postoperative[tiab]

OR iatrogenic[tiab]--))

## Appendix 2 PRISMA 2020 reporting and study selection

The identification and selection of studies were conducted and reported in accordance with the PRISMA 2020 statement, adapted for a bibliometric/mapping review in which the primary unit of analysis is bibliographic metadata rather than aggregated clinical outcomes. Searches were performed in PubMed (MEDLINE), Scopus, the Cochrane Library, and JSTAGE (January 1977 to February 2025). Records retrieved across databases were merged and de-duplicated prior to screening. Titles and abstracts were screened for relevance to acute conus medullaris syndrome (ACMS) and conus-related postoperative complications following lumbar spine procedures. Full texts were then assessed for eligibility.

Records identified from databases (*n* = 342); duplicates removed before screening (*n* = 46); records screened (*n* = 296); records excluded at title/abstract stage (*n* = 198); reports sought for retrieval (*n* = 98); reports not retrieved (*n* = 0); reports assessed for eligibility (*n* = 98); reports excluded with reasons (*n* = 52: not ACMS/wrong condition *n* = 22; not post-lumbar procedure context *n* = 14; duplicate content/overlap *n* = 8; non-eligible publication type *n* = 8); studies included in the final bibliometric corpus (*n* = 46).
